# Acrodictys-like wood decay fungi from southern China, with two new families *Acrodictyaceae* and *Junewangiaceae*

**DOI:** 10.1038/s41598-017-08318-x

**Published:** 2017-08-11

**Authors:** Ji Wen Xia, Ying Rui Ma, Zhuang Li, Xiu Guo Zhang

**Affiliations:** Shandong Provincial Key Laboratory for Biology of Vegetable Diseases and Insect Pests, College of Plant Protection, Shandong Agricultural University, Taian, 271018 Shandong China

## Abstract

During our continuous survey (2012*–*2016) of saprobic hyphomycetes from dead branches in the forest ecosystems of southern China, we collected several acrodictys-like species. Acrodictys-like species are characterized by darkly pigmented and muriform conidia produced from holoblastic conidiogenous cells on macronematous, mononematous, cylindrical and unbranched or infrequently branched conidiophores. Phylogenetic analyses of ncLSU, ncSSU, ITS and *tub2* sequence data lead us to propose two novel families in Sordariomycetes, *Acrodictyaceae* and *Junewangiaceae*. In addition, a new species, *Acrodictys hainanensis*, two new combinations, *Junewangia queenslandica* and *Distoseptispora martinii*, three new Chinese records, *Acrodictys liputii*, *A. peruamazonensis* and *Junewangia sphaerospora* are introduced. Two names, *Acrodictys globulosa* and *A. malabarica*, are resurrected.

## Introduction

Acrodictys-like species are saprobic on dead branches and have a worldwide distribution. *Acrodictys* was introduced by Ellis with *A. bambusicola* as the type species^1^. Subsequently, more than 40 species have been referred to the genus. Baker *et al*. and Baker & Morgan-Jones refined the generic concept of *Acrodictys* in a strict sense as conidiophore commonly indeterminate and proliferating percurrently, successive terminal proliferations lageniform to doliiform, conidia muriform, usually with vertical-longitudinal septa in the middle cells and several parallel-transversal septa^2–4^. They established three other genera i.e. *Junewangia*, *Rhexoacrodictys* and *Pseudoacrodictys* for accommodating some *Acrodictys sensu lato* species based on conidial morphology, conidiogenesis and type of conidial secession. Gams *et al*. and Zhao *et al*. erected *Bhatia* and *Ramoacrodictys*, respectively, to accommodate *A. malabarica*, on the basis of branched conidiophores and distinctively ornamented dictyoconidia^5, 6^. Seifert *et al*. accepted *Bhatia* as the valid name^7^.

In previous studies on acrodictys-like species, identification has been based on morphology and no acrodictys-like species has been subjected to molecular phylogenetic analysis^8–26^. Maharachchikumbura *et al*. provided a natural classification backbone for Sordariomycetes^27, 28^. The resulting data allows us to place Acrodictys-like species in a natural taxonomic framework^27–29^. In this study, we revisit the acrodictys-like species, and formally introduce two new families *Acrodictyaceae* and *Junewangiaceae* according to the molecular data. In addition, a new species, *Acrodictys hainanensis*, two new combinations, *Junewangia queenslandica* and *Distoseptispora martinii*, three new Chinese records, *Acrodictys liputii*, *A. peruamazonensis* and *Junewangia sphaerospora* are introduced. Two names, *Acrodictys globulosa* and *A. malabarica*, are resurrected.

## Results

### Phylogeny

The final concatenated alignment contained 51 ingroup taxa with a total of 3169 characters including gaps (559 for LSU, 1538 for SSU, 638 for ITS, 434 for *tub2*) of which 842 were unique site patterns (241 for LSU, 142 for SSU, 304 for ITS, 155 for *tub2*), with *Botryotinia fuckeliana* and *Dothidea sambuci* as the outgroup taxa. The general time reversible model with inverse gamma rates (GTR + I + G) was determined to be the best for all four loci by MrModeltest. The LSU, SSU, ITS and *tub2* sequence datasets did not show any conflicts in the tree topologies for the 70% reciprocal bootstrap trees, which allowed to combine the four loci for the multi-locus analysis.

**Figure 1:**
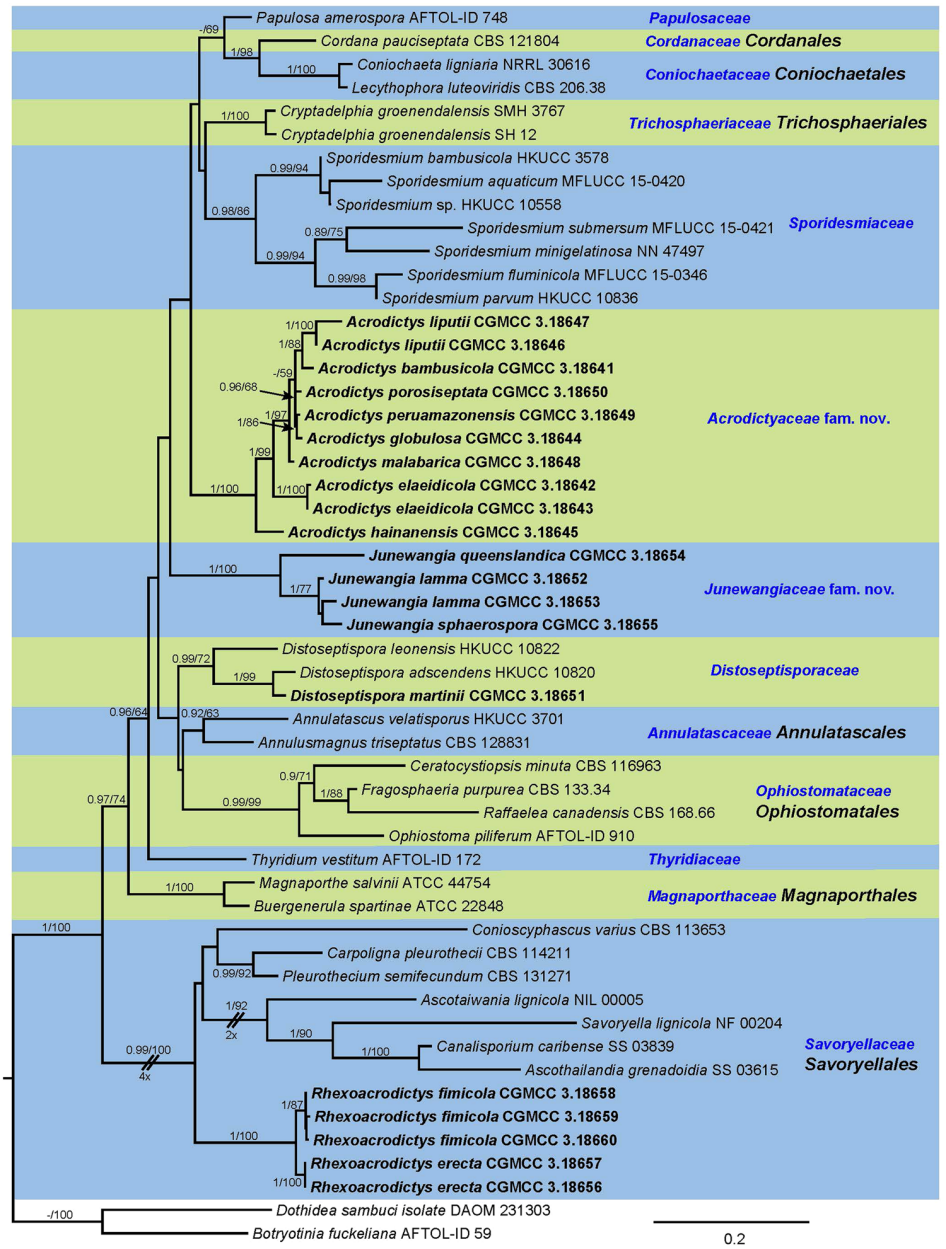
Phylogenetic tree inferred from a Maximum likelihood analysis based on a concatenated alignment of LSU, SSU, ITS and *tub2* sequences of 51 strains representing Sordariomycetes. Only high branch support is shown at the nodes, maximum likelihood bootstrap support (MLBS ≥ 50%) and Bayesian posterior probability (PP ≥ 85%). Taxa in bold refer to newly introduced sequence. Some branches were shortened to fit them to the page – these are indicated by two diagonal lines with the number of times a branch was shortened indicated next to the lines. The tree is rooted to *Botryotinia fuckeliana* and *Dothidea sambuci*.

For the multi-locus analyses, a total of 1025 trees were sampled after the burn-in with a stop value of 0.01. The topology of the BI tree confirmed that of ML tree for the distinctions of 13 well supported monophyletic clades, and therefore only the ML consensus tree with Bayesian posterior probabilities (BPP) and RAxML bootstrap support (MLBS) values are indicated in Fig. 1.

The phylogenetic tree delimited thirteen families, two of which are described here as new (*Acrodictyaceae* and *Junewangiaceae*), and eleven previously included families namely *Annulatascaceae*, *Coniochaetaceae*, *Cordanaceae*, *Distoseptisporaceae*, *Magnaporthaceae*, *Ophiostomataceae*, *Papulosaceae*, *Sporormiaceae*, *Savoryellaceae*, *Trichosphaeriaceae* and *Thyridiaceae*.

### Taxonomy

***Acrodictyaceae*** J.W. Xia & X.G. Zhang, *fam. nov*. – MycoBank MB 818894.

*Etymology*. According to the type genus, *Acrodictys*.

*Type genus*. *Acrodictys* M.B. Ellis, Mycol. Pap. 79: 6. 1961.

Colonies on the substratum superficial, effuse, hairy or velvety, black. Mycelia mostly immersed, composed of branched, septate, smooth, pale brown hyphae. Sexual morph: Undetermined. Asexual morph: Hyphomycetous. Conidiophores macronematous, mononematous, septate, single or in groups, erect, straight or flexuous, smooth, pale brown to brown, cylindrical, robust at the base. Conidiogenous cells monoblastic, integrated, determinate, terminal, cylindrical. Conidia acrogenous, solitary, dry, brown to dark brown, obovoid to pyriform, muriform, conidial secession schizolytic.

Notes – A new family *Acrodictyaceae* is hereby introduced to accommodate species with obovoid to pyriform, muriform conidia and their asexual morphs that form a monophyletic clade in the class *Sordariomycetes* (Fig. 1). No sexual morph is known for this family.

***Acrodictys*** M.B. Ellis, Mycol. Pap. 79: 6. 1961.

*Type species*. *Acrodictys bambusicola* M.B. Ellis, Mycol. Pap. 79: 6. 1961.

Colonies on the substratum superficial, effuse, hairy or velvety, black. Mycelia mostly immersed, composed of branched, septate, smooth, pale brown hyphae. Sexual morph: Undetermined. Asexual morph: Hyphomycetous. Conidiophores macronematous, frequently bulbous at the base, mononematous, erect, straight or flexuous, thick-walled, smooth, typically indeterminate and proliferating percurrently. Conidiogenous cells monoblastic, integrated, terminal. Conidia solitary, dry, acrogenous, obovoid to pyriform, muriform; septa transverse and longitudinal, the transverse septa typically spanning the whole conidial width, the longitudinal septa typically incomplete, short; mid to dark-blackish brown, often with a graduation from lighter at the base to darker toward the apex, smooth, narrowly truncate at the base, seceding schizolytically.

Notes — *Acrodictys* was introduced by Ellis with *A. bambusicola* M.B. Ellis as the type species^1^. The holotype was deposited in Herb. IMI. No culture derived from the type collection exists.

**Figure 2:**
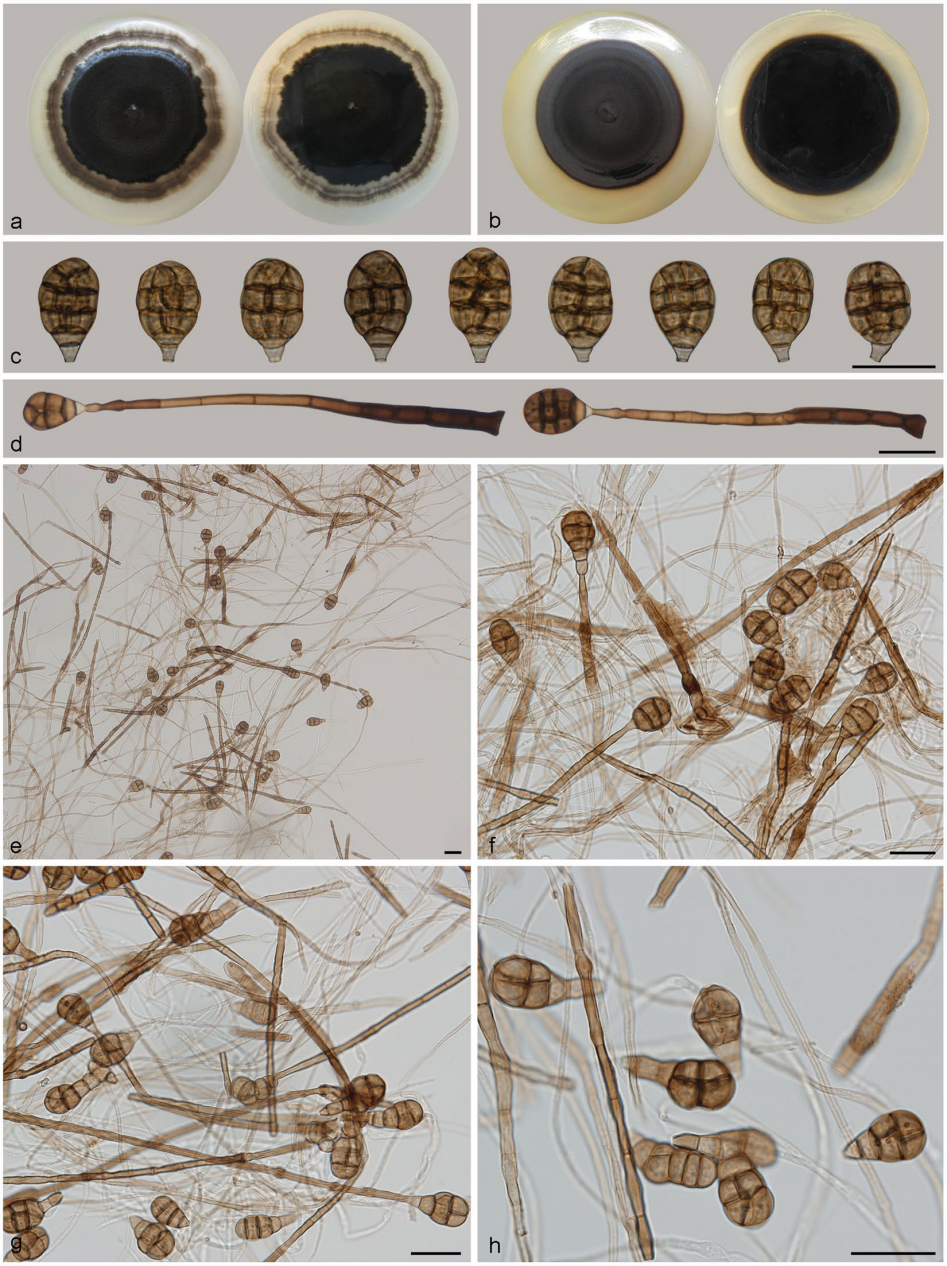
Acrodictys bambusicola. (**a**) Colony on PDA (surface and reverse). (**b**) Colony on MEA (surface and reverse). (**c**,**d**) From natural substrate (HSAUP H9510): (**c**) Conidia, (**d**) Conidiophores with conidia. (**e**–**h**) From PDA (CGMCC 3.18641): Conidiophores and conidia. — Scale bars = 20 μm.

***Acrodictys bambusicola*** M.B. Ellis, Mycol. Pap. 79: 6. 1961. — Fig. 2.

Conidiophores macronematous, mononematous, erect, unbranched, straight or flexuous, thick-walled, smooth, dark brown at the base, narrower and paler toward the apex, 3–7-septate, 128–150 *μ*m long, 4–7 *μ*m wide. Conidiogenous cells integrated, terminal, indeterminate with 1–3 percurrent extensions, cylindrical, lageniform to doliiform, subhyaline to pale brown, smooth, monoblastic. Conidia solitary, muriform, broadly clavate, obovoid to pyriform, usually with 3–4 transverse septa and a few longitudinal septa, slightly constricted at the septa, 20–29 × 12.5–22.5 μm, pale brown at the basal two cells and brown at the other part, basal cell obconical, truncate at base.

Culture characteristics – Colonies on PDA, 75–80 mm diam after 14 d at 25 °C, mycelium sparse, dark brown to black; reverse concolourous. Colonies on MEA, 55–60 mm diam after 14 d at 25 °C, margin regular, dark brown to black; reverse concolourous.

*Materials examined*. China, Hainan Province, Lingshui, Diaoluoshan National Forest Park, 18°42′N, 108°52′E, 1499 m elevation, on dead branches of an unidentified bamboo, 22 Apr. 2014, Jianmei Gao, reference specimen designated here HSAUP H9510 (=HMAS 245623), living culture CGMCC 3.18641.

Notes — *Acrodictys bambusicola* is the type species of *Acrodictys* Ellis^1^. Our specimen is similar to the holotype material from Venezuela, except for the slightly lighter colour of conidia in the Chinese collection. It would be unwise to epitypity this species with the China collection as they are from different continents. We therefore designate our collection as a reference specimen of *A. bambusicola* so that further work on this taxon can be carried out.

**Figure 3:**
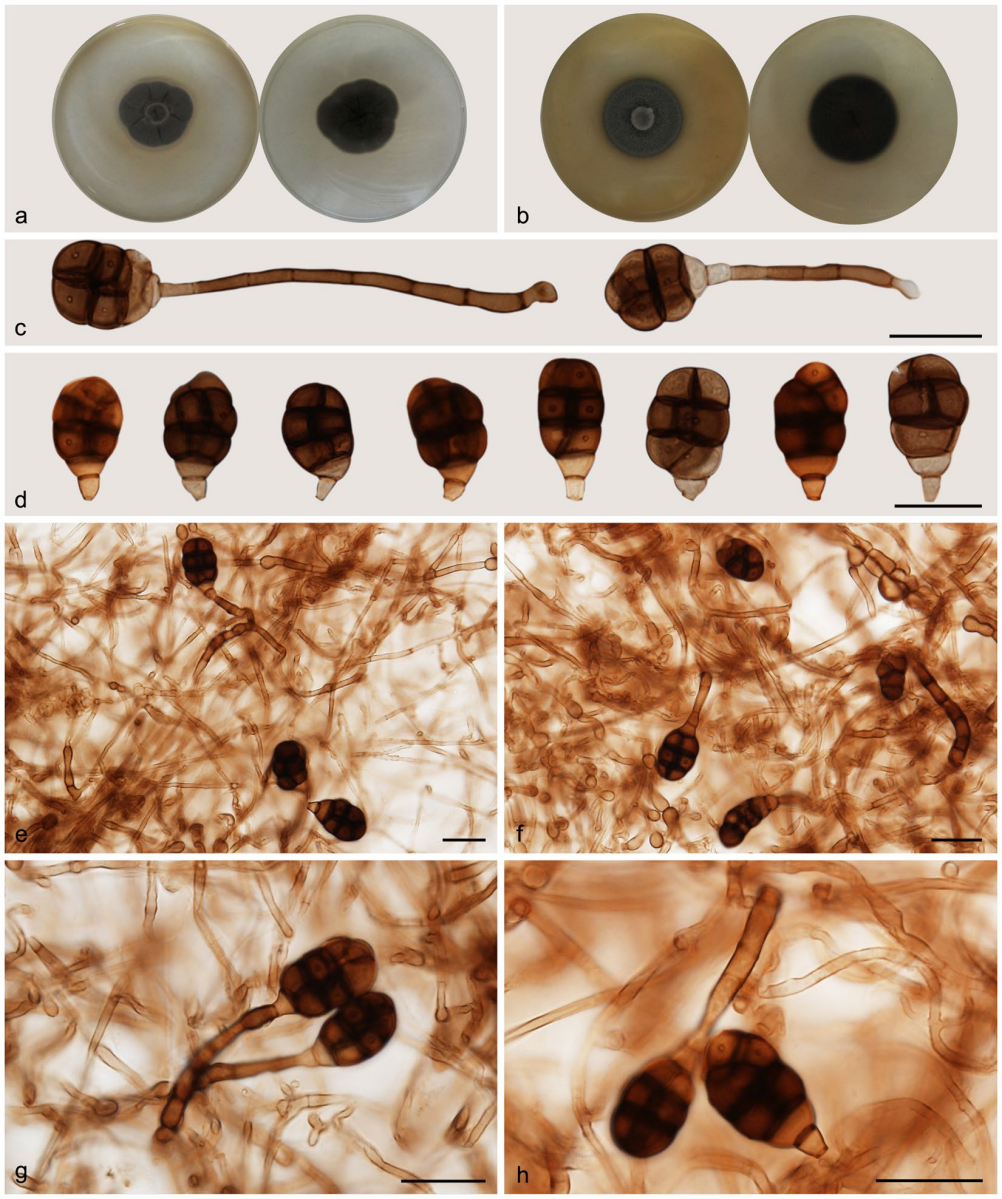
Acrodictys elaeidicola. (**a**) Colony on PDA (surface and reverse). (**b**) Colony on MEA (surface and reverse). (**c**,**d**) From natural substrate (HSAUP H5528): (**c**) Conidiophores with conidia, (**d**) Conidia. (**e**–**h**) From PDA (CGMCC 3.18642): Conidiophores and conidia. — Scale bars = 20 μm.

***Acrodictys elaeidicola*** M.B. Ellis, Mycol. Pap. 79: 7. 1961. — Fig. 3.

Conidiophores macronematous, mononematous, erect, unbranched, straight or flexuous, thick-walled, smooth, dark brown at the base, paler toward the apex, septate, 25–89 × 2.5–4 *μ*m. Conidiogenous cells monoblastic, integrated, terminal, determinate, cylindrical, subhyaline to pale brown, smooth. Conidia solitary, muriform, obovoid to pyriform, 22–31 × 14–22 *μ*m, usually with 3–4 transverse septa and 1–3 longitudinal or oblique septa, the latter mainly subdividing the upper portion of the conidium, with the lower two cells remaining undivided, slightly constricted at the septa, brown above and hyaline to pale brown in the lower half, narrowly truncate at the base.

Culture characteristics – Colonies on PDA, 25–30 mm diam after 14 d at 25 °C, mycelium sparse, dark brown, pale brown near the colony margin; reverse concolourous. Colonies on MEA, 45–50 mm diam after 14 d at 25 °C, margin regular, dark brown, pale brown near the colony margin; reverse concolourous.

*Materials examined*. China, Hainan Province, Changjian, Bawangling National Forest Park, 18°57′N, 109°03′E, 1099 m elevation, on dead branches of an unidentified broadleaf tree, 20 Apr. 2010, Jian Ma, reference specimen designated here HSAUP H5528 (=HMAS 245627), HSAUP H5536 (=HMAS 245628), living culture CGMCC 3.18642, CGMCC 3.18643.

Notes — Compared with the specimens on natural substrate, the conidiophores produced in culture were shorter, but the conidial septation and shape are similar. Among species of *Acrodictys*, *A. elaeidicola* is similar to *A. bambusicola* in conidial shape. However, conidia of *A. elaeidicola* have fewer secondary longitudinal or oblique septa^3^. Conidiogenous cells in the type collection of *A. elaeidicola* have conspicuous percurrent extensions while percurrent extensions are not visible in our Chinese material. We consider this to be a minor difference. Given the similarity in conidial shape, pigmentation and number of septa, we are confident to identify above Chinese collection as *A. elaeidicola*.

**Figure 4:**
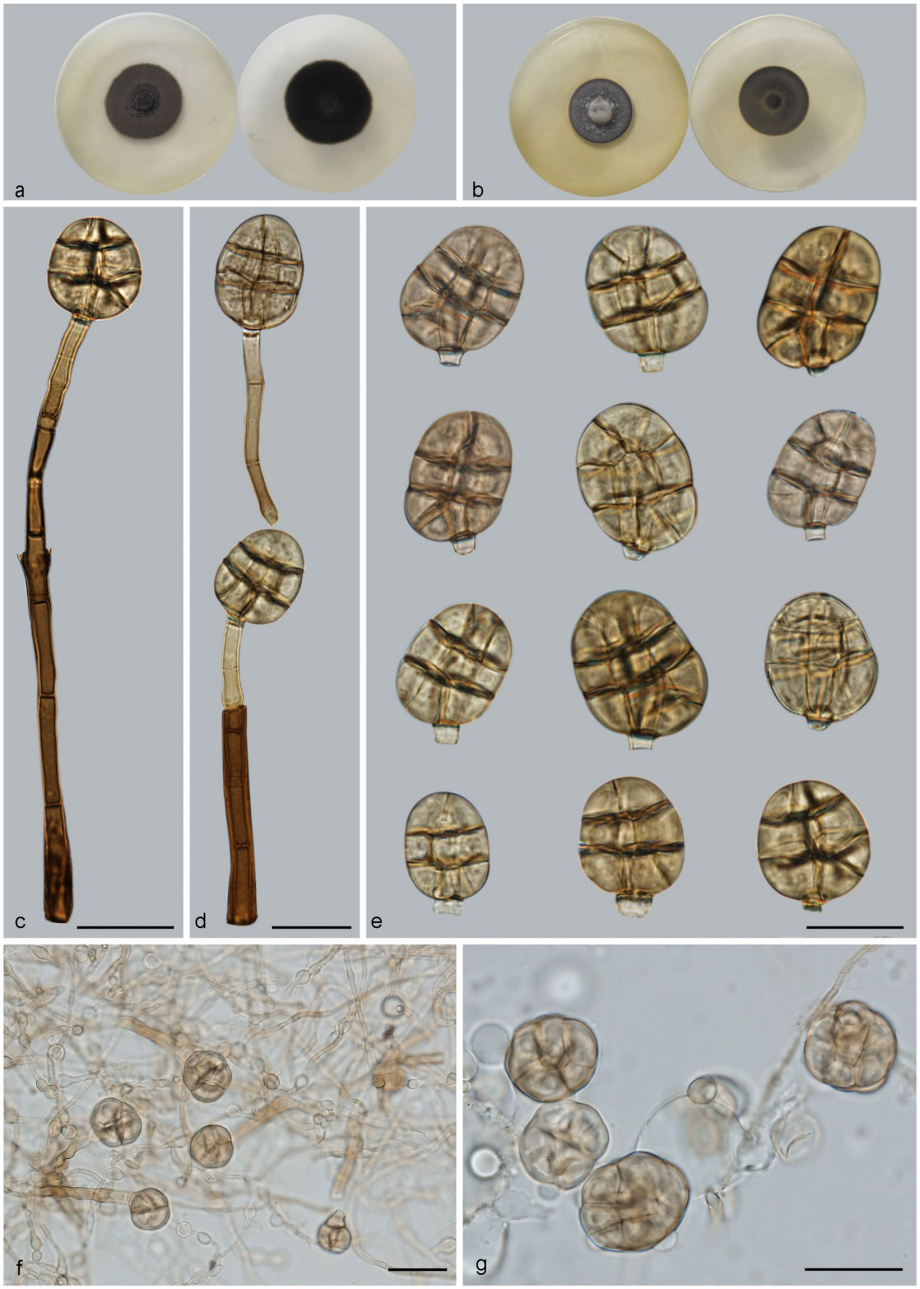
Acrodictys globulosa. (**a**) Colony on PDA (surface and reverse). (**b**) Colony on MEA (surface and reverse). (**c**–**e**) From natural substrate (HSAUP H4696): (**c**,**d**) Conidiophores with conidia, (**e**) Conidia. (**f**,**g**) From PDA (CGMCC 3.18644): Conidiophores and conidia. — Scale bars = 20 μm.

***Acrodictys globulosa*** (Tóth) M.B. Ellis, Mycol. Pap. 103: 34. 1965. — Fig. 4.

Basionym: *Monodictys globulosa* Tóth, Anal. Nat. Tabl. Univ. 54: 183. 1962.

≡ *Junewangia globulosa* (Tóth) W.A. Baker & Morgan-Jones, Mycotaxon 81: 308. 2002.

Conidiophores macronematous, mononematous, erect, single or in clusters of two or three, unbranched, straight or flexuous, thick-walled, smooth, dark brown at the base, paler toward the apex, septate, 45–129 × 3.5–6 *μ*m. Conidiogenous cells monoblastic, integrated, indeterminate with 0–3 successive percurrent extensions, subhyaline to pale brown, smooth, terminal, widely truncate at the apex following conidium disarticulation. Conidia solitary, muriform, subglobose, 20.5–31.5 × 16.5–25 *μ*m, with 2–(3) transverse septa and some longitudinal or oblique septa, pale to cinnamon brown, with a distinctly protuberant, broadly cuneate basal cell. Conidial secession schizolytic.

Culture characteristics — Colonies on PDA, 35–40 mm diam after 14 d at 25 °C, brown to dark brown, mycelium sparse; reverse concolorous. Colonies on MEA, 25–30 mm diam after 14 d at 25 °C, margin regular, brown; reverse concolorous.

*Materials examined*. China, Yunnan Province, Xishuangbanna, Menglun Nature Reverse, 21°27′N, 100°25′E, 552 m elevation, on dead branches of unidentified broadleaf tree, 22 Nov. 2015, Yingrui Ma, reference specimen designated here HSAUP H4696 (=HMAS 245621), living culture CGMCC 3.18644.

Notes — Baker *et al*. transferred *A. globulosa* to *Junewangia* based on its subglobose conidia^3^. However, the conidiophores of *A. globulosa* are longer and have fewer percurrent extensions than *Junewangia* species. The combined LSU-SSU-ITS-*tub2* phylogenies also confirm that *A. globulosa* clustered in *Acrodictys* (Fig. 1). The combination of the morprathhological features and phylogenetic analyses indicate that *A. globulosa* belongs to *Acrodictys* rather than *Junewangia*.

**Figure 5:**
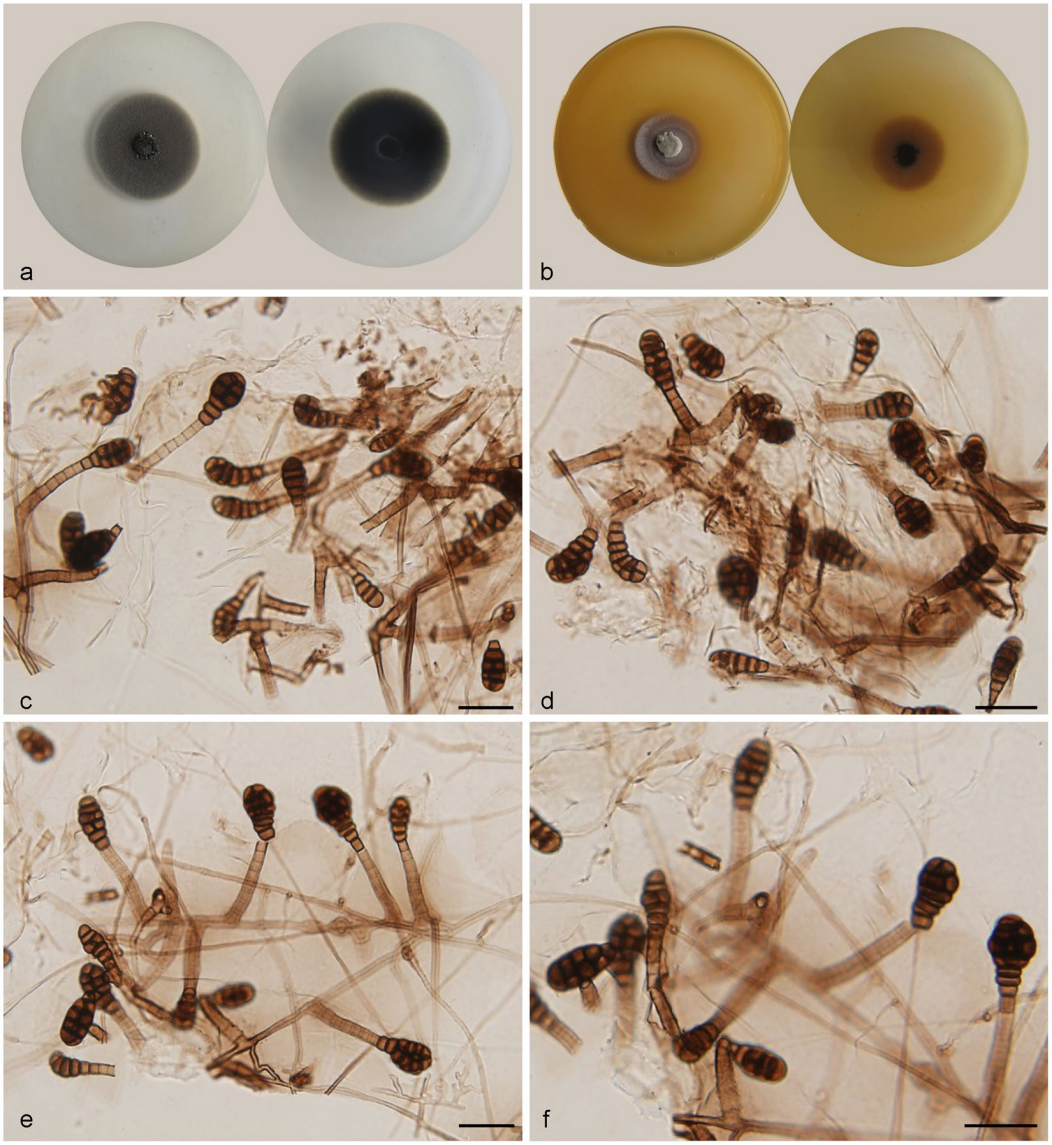
Acrodictys hainanensis. (**a**) Colony on PDA (surface and reverse). (**b**) Colony on MEA (surface and reverse). (**c**–**f**) From PDA (CGMCC 3.18645): Conidiophores and conidia. — Scale bars = 20 μm.

***Acrodictys hainanensis*** J.W. Xia & X.G. Zhang, *sp. nov*. – MycoBank MB 818895; — Fig. 5.

*Etymology*. In reference to the type locality.

Conidiophores up to 35 *μ*m long in culture, macronematous, mononematous, erect, unbranched, pale brown, septate. Conidiogenous cells monoblastic, integrated, terminal. Conidia oblong to obovoid, 15–22 × 7–13 *μ*m, muriform, usually with 3–5 transverse septa and several longitudinal or oblique septa, pale brown; basal cell truncate, cylindrical, pale brown. Conidial secession schizolytic.

Culture characteristics – Colonies on PDA, 35–45 mm diam after 14 d at 25 °C, surface dark brown, mycelium sparse; reverse blackish. Colonies on MEA, 30–40 mm diam after 14 d at 25 °C, margin regular, surface dark brown, reverse blackish.

*Materials examined*. China, Hainan Province, Lingshui, Diaoluoshan National Forest Park, 18°42′N, 108°52′E, 1499 m elevation, on dead branches of an unidentified broadleaf tree, 22 Apr. 2013, Xiangyu Li, holotype HMAS 245624, ex-holotype living culture CGMCC 3.18645.

Notes — *Acrodictys hainanensis* is morphologically similar to *A. peruamazonensis*, but clearly differs in the size of conidia (15–22 × 7–13 *μ*m vs. 23–34 × 18–22 *μ*m). The two species are phylogenetically distinct (Fig. 1). For these reasons and we introduce *A. hainanensis* as a new species.

**Figure 6:**
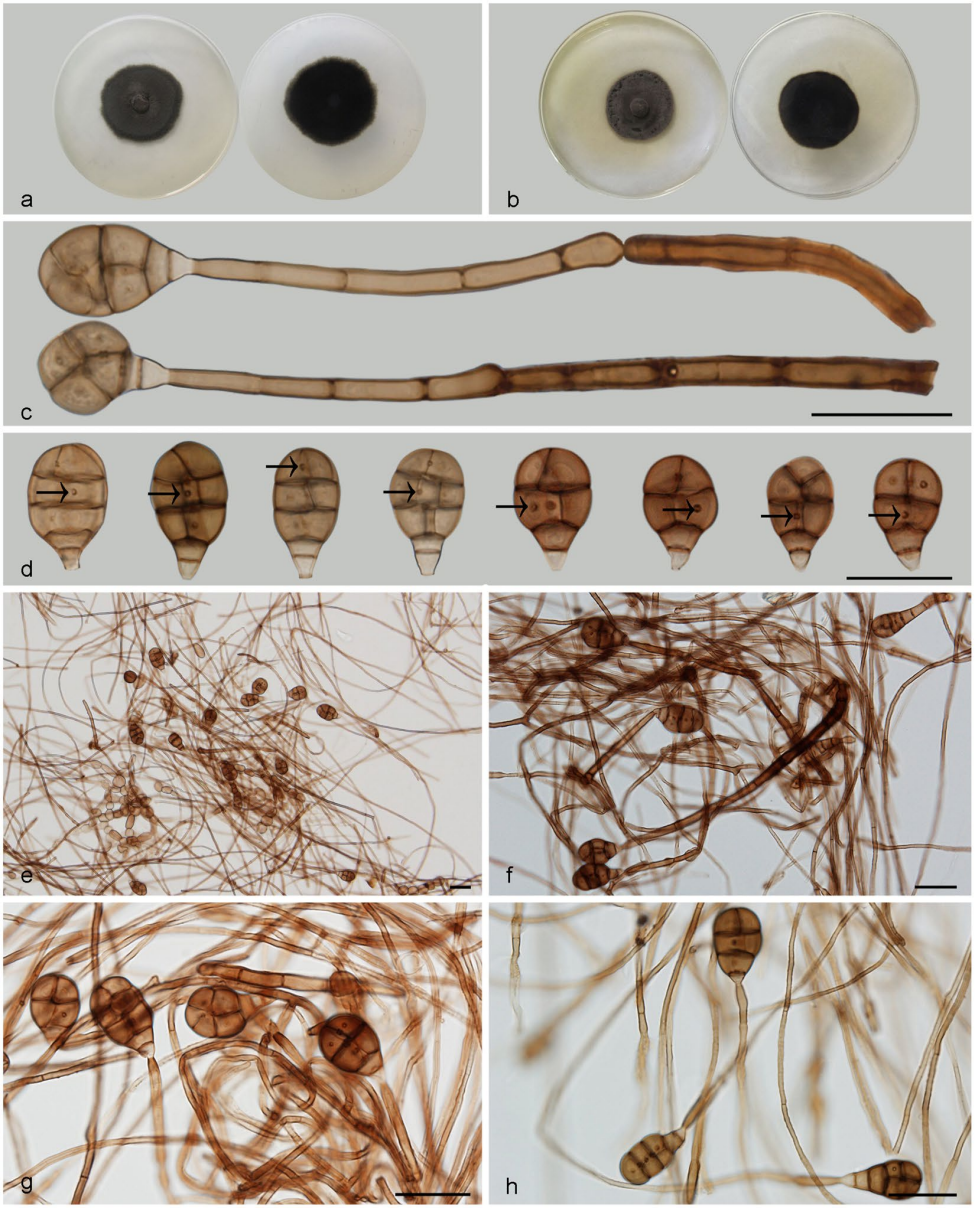
Acrodictys liputii. (**a**) Colony on PDA (surface and reverse). (**b**) Colony on MEA (surface and reverse). (**c**,**d**) From natural substrate (HSAUP H1883): (**c**) Conidiophores with conidia, (**d**) Conidia. (**e**–**h**) From PDA (CGMCC 3.18646): Conidiophores and conidia. — Scale bars = 20 μm.

***Acrodictys liputii*** L. Cai, K.Q. Zhang, McKenzie, W.H. Ho & K.D. Hyde, Nova Hedwigia 75 (3–4): 526, 2002. — Fig. 6.

Conidiophores macronematous, mononematous, erect, unbranched, solitary, pale brown to brown at the base, subhyaline to pale yellow at the apex, smooth, with walls thickened especially towards the base, 4–9-septate, 34–120 × 4–6 *μ*m. Conidiogenous cells monoblastic, integrated into the apex of the conidiophores, terminal, smooth, cylindrical, subhyaline to pale yellow, truncate. Conidia acrogenous, solitary, dry, terminal, subglobose, smooth, pale brown, 18–26 × 12–15 *μ*m, mostly with 3–4 parallel transverse septa and 2 perpendicular longitudinal septa, with conspicuous pores in the septa.

Culture characteristics — Colonies on PDA, 35–40 mm diam after 14 d at 25 °C, mycelium sparse, dark brown to blackish; reverse concolorous. Colonies on MEA, 35–40 mm diam after 14 d at 25 °C, margin regular, dark brown; reverse concolorous.

*Materials examined*. China, Hainan Province, Lingshui, Diaoluoshan National Forest Park, 18°42′N, 108°52′E, 1499 m elevation, on dead branches of an unidentified broadleaf tree, 22 Apr. 2014, Xiangyu Li, reference specimen designated here HSAUP H1883 (=HMAS 245629), HSAUP H2137 (=HMAS 245617), living culture CGMCC 3.18646, CGMCC 3.18647.

Notes — Our specimens are compared to the original description, which was based on material collected from the Philippines, except for their slightly shorter conidiophores (34–120 *μ*m vs 75–220 μm)^30^. This is the first report of this species from China.

**Figure 7:**
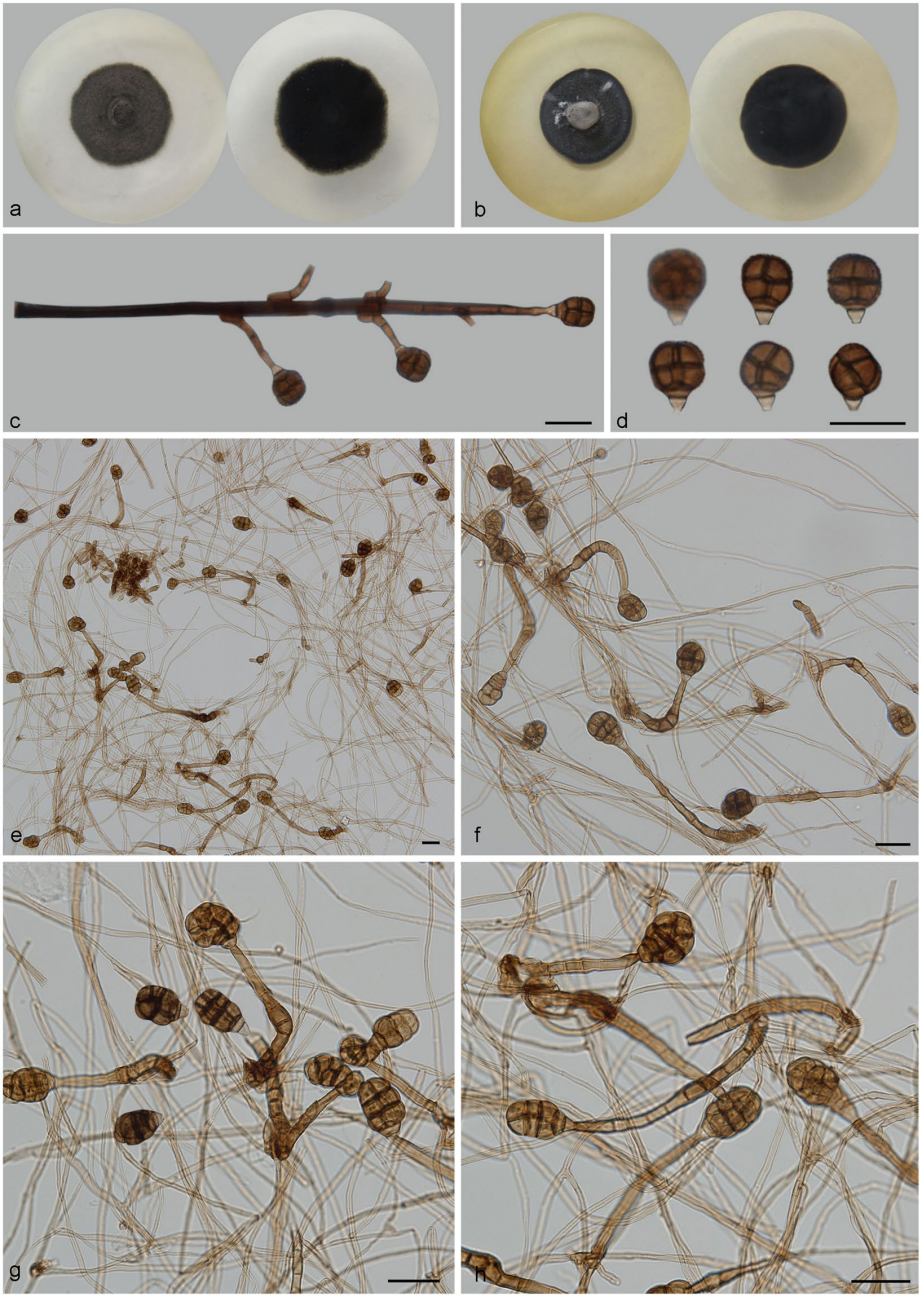
Acrodictys malabarica. (**a**) Colony on PDA (surface and reverse). (**b**) Colony on MEA (surface and reverse). (**c**,**d**) From natural substrate (HSAUP H9509): (**c**) Conidiophores with fertile branches and conidia, (**d**) Conidia. (**e**–**h**) From PDA (CGMCC 3.18648): Conidiophores and conidia. — Scale bars = 20 μm.

***Acrodictys malabarica*** Subram. & Bhat, Kavaka 15: 41. 1989. — Fig. 7.

Basionym: *Bhatia malabarica* (Subram. & Bhat) W.A. Baker & Morgan-Jones, Mycotaxon 110: 93. 2009.

=*Ramoacrodictys malabarica* (Subram. & Bhat) G.Z. Zhao, Sydowia 61: 355. 2009.

Conidiophores macronematous, mononematous, erect, simple to multi-branched, straight or slightly flexuous, thick-walled, smooth, dark brown at the base, tapering and paler towards the apex, septate, 150–280 *μ*m long, 5.5–10 *μ*m wide, with fertile branches arranged in verticils along the main axis; conidiophore branches usually pale brown, smooth, thin-walled, truncate at the apex, 13–56 × 2–4.5 *μ*m. Conidiogenous cells monoblastic, integrated, terminal, determinate, cylindrical, lageniform to doliiform, subhyaline to light brown, smooth, terminal on stipe and branches. Conidia solitary, acrogenous, broadly ellipsoidal or obovoid, 18–24 × 12.5–16 *μ*m, muriform, septa sometimes cruciate, usually with 1–3 transverse septa and a few longitudinal and oblique septa, constricted at the septa, brown to dark brown. Basal 1–2 cells protuberant, subhyaline to pale brown, cuneiform, truncate at base. Conidial secession schizolytic.

Culture characteristics – Colonies on PDA, 40–45 mm diam after 14 d at 25 °C, mycelium sparse, dark brown to black; reverse blackish. Colonies on MEA, 35–40 mm diam after 14 d at 25 °C, margin regular, dark brown to black; reverse blackish.

*Materials examined*. China, Hainan Province, Lingshui, Diaoluoshan National Forest Park, 18°42′N, 108°52′E, 1499 m elevation, on dead branches of an unidentified bamboo, 22 Apr. 2014, Jianmei Gao, reference specimen designated here HSAUP H9509 (=HMAS 245619), living culture CGMCC 3.18648.

Notes — *Acrodictys malabarica* was excluded from *Acrodictys* because of its multi-branched conidiophores, non-proliferating conidiogenous cells^5, 6^, however, the pyriform or obovoid conidia with muriform septa indicate its similarity to some *Acrodictys* species including *A. bambusicola*, *A. atroapicula* C.J.K. Wang & B. Sutton and *A. elaeidicola* M.B. Ellis. In addition, in the phylogenetic tree (Fig. 1), *A. malabarica*, *A. bambusicola*, *A. elaeidicola*, *A. globulosa*, *A. hainanensis*, *A. liputii*, *A. peruamazonensis* and *A. porosiseptata* grouped together within the *Acrodictys* clade.

**Figure 8:**
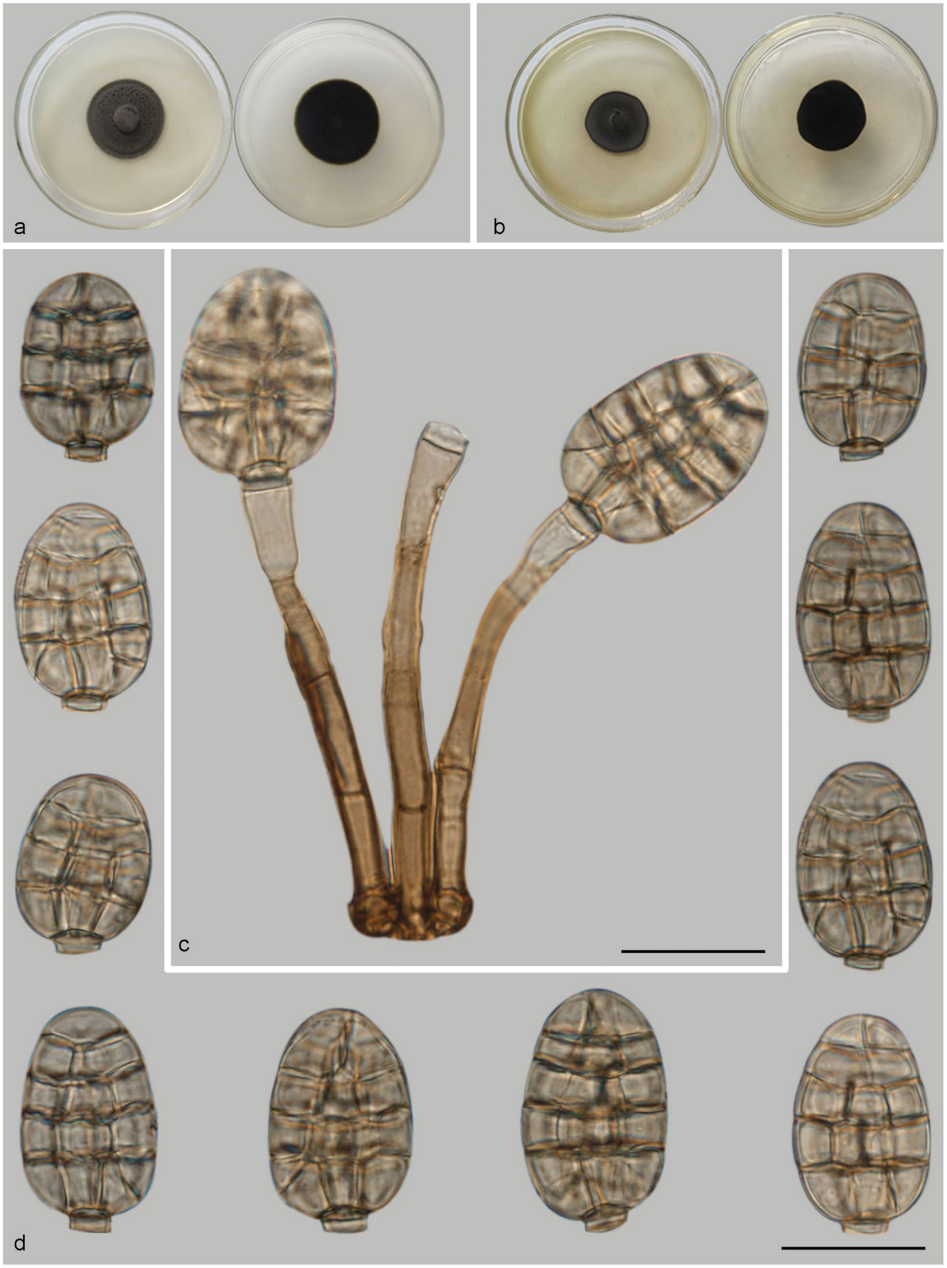
Acrodictys peruamazonensis. (**a**) Colony on PDA (surface and reverse). (**b**) Colony on MEA (surface and reverse). (**c**,**d**) From natural substrate (HSAUP H4694): (**c**) Conidiophores with conidia, (**d**) Conidia. — Scale bars = 20 μm.

***Acrodictys peruamazonensis*** Matsush., Matsushima Mycological Memoirs 7: 42. 1993. — Fig. 8.

Conidiophores macronematous, mononematous, erect, in clusters of two or three, rarely single, unbranched, straight or flexuous, thick-walled, smooth, dark brown at the base, subhyaline towards the apex, 2–5 septate, 35–70 × 3.5–5 *μ*m. Conidiogenous cells monoblastic, integrated, terminal, with 0–2 percurrent extensions, pale brown to subhyaline, smooth, widely truncate at the apex following conidium dehiscence. Conidia solitary, cylindrical to ellipsoidal, 23–34 × 18–22 μm, muriform with 3–4 transverse septa and some longitudinal or oblique septa, pale to cinnamon brown. Basal cell distinct. Conidial secession schizolytic.

Culture characteristics – Colonies on PDA, 30–35 mm diam after 14 d at 25 °C, brown to dark brown, mycelium sparse; reverse blackish. Colonies on MEA, 25–30 mm diam after 14 d at 25 °C, margin regular, brown to dark brown; reverse blackish.

*Materials examined*. China, Yunnan Province, Xishuangbanna, Menglun Nature Reverse, 21°27′N, 100°25′E, 552 m elevation, on dead branches of unidentified broadleaf tree, 20 Nov. 2015, Yingrui Ma, reference specimen designated here HSAUP H4694 (=HMAS 245620), living culture CGMCC 3.18649.

Notes — Our specimen compares well with the original description, which was based on material from Peru, but in the Chinese collection conidiophores are more highly clustered and shorter (35–70 μm vs 50–100 μm). Despite these minor differences, we believe they are the same species. This is the first report of this species from China.

**Figure 9:**
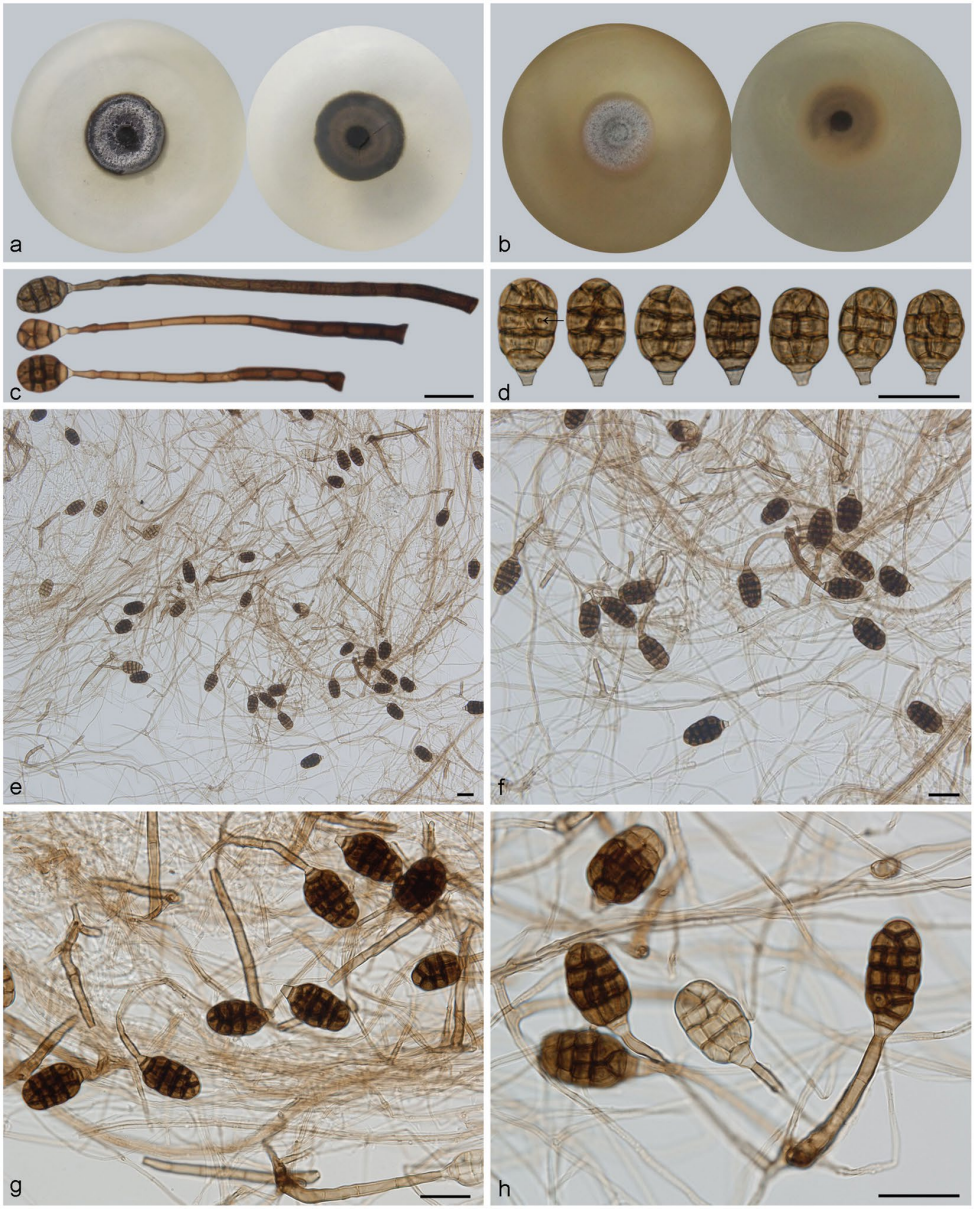
Acrodictys porosiseptata. (**a**) Colony on PDA (surface and reverse). (**b**) Colony on MEA (surface and reverse). (**c**,**d**) From natural substrate (HSAUP H4698): (**c**) Conidiophores with conidia, (**d**) Conidia. (**e**–**h**) From PDA (CGMCC 3.18650): Conidiophores and conidia. — Scale bars = 20 μm.

***Acrodictys porosiseptata*** G.Z. Zhao, Mycological Progress 10: 74. 2011. — Fig. 9.

Conidiophores macronematous, mononematous, single or in groups of two or three, erect, unbranched, straight or flexuous, thick-walled, smooth, dark brown at the base, narrower and paler toward the apex, 4–10-septate, 40–175 × 3.5–6 μm. Conidiogenous cells monoblastic, integrated, terminal, indeterminate with 0–3 successive percurrent extensions, cylindrical, lageniform to doliiform, subhyaline to pale brown, smooth. Conidia solitary, broadly clavate, obovoid to pyriform, 20–36.5 × 13.5–15.5 μm, muriform usually with 4–(5) transverse septa, 3–4 longitudinal septa and a few oblique septa, transverse septa sometimes thick and darkly pigmented, slightly constricted at the septa, conspicuous pores appearing at the surface of the conidia. Basal cell protruding, obconical, pale brown, truncate at base. Conidial secession schizolytic.

Culture characteristics – Colonies on PDA, 25–30 mm diam after 14 d at 25 °C, brown, mycelium sparse, grey; reverse brown. Colonies on MEA, 25–30 mm diam after 14 d at 25 °C, margin regular, white to light grey; reverse concolorous.

*Materials examined*. China, Yunnan Province, Xishuangbanna, Menglun Nature Reserve, 21°27′N, 100°25′E, 552 m elevation, on dead branches of an unidentified bamboo, 22 Nov. 2015, Yingrui Ma, reference specimen designated here HSAUP H4698 (=HMAS 245618), living culture CGMCC 3.18650.

Notes — *Acrodictys porosiseptata* is characterized by its long conidiophores, conspicuous swellings that mark the extensions of the conidiogenous cells, and subglobose or pyriform muriform conidia that have conspicuous septal pores. This suite of characters distinguishes this species from morphologically comparable species including *A. liputii* L. Cai, *A. balladynae* (Hansf.) M.B. Ellis, *A. elaeidicola* M.B. Ellis and *A. similes* Hol.-Jech^31^.

***Junewangiaceae*** J.W. Xia & X.G. Zhang, *fam. nov*. – MycoBank MB 818897.

*Etymology*. According to the type genus, *Junewangia*.

*Type genus*. *Junewangia* W.A. Baker & Morgan-Jones, Mycotaxon 81: 307. 2002.

Colonies effuse, dark brown, hairy. Mycelia partly superficial, partly immersed in the substrate. Sexual morph: Undetermined. Asexual morph: Hyphomycetous. Conidiophores macronematous, mononematous, erect, unbranched, straight or flexuous, cylindrical, thick-walled, smooth, septate, brown at the base, colorless towards the apex. Conidiogenous cells monoblastic, integrated, terminal, cylindrical, brown, pale brown or subhyaline, smooth or verrucose; collarette narrow or flaring. Conidia solitary, dry, apical, simple, smooth, typically oval, ellipsoidal to spherical, brown to dark brown.

Notes — A new family, *Junewangiaceae*, is introduced to accommodate species having oval, ellipsoidal to spherical conidia. The family is monotypic for the genus *Junewangia*. No sexual morph is known for this family.

***Junewangia*** W.A. Baker & Morgan-Jones, Mycotaxon 81: 307. 2002.

*Type species*. *Junewangia sphaerospora* W.A. Baker & Morgan-Jones, Mycotaxon 81: 312. 2002.

Colonies effuse, dark brown, hairy. Mycelia partly superficial, partly immersed in the substrate. Sexual morph: Undetermined. Asexual morph: Hyphomycetous. Conidiophores macronematous, mononematous, erect, unbranched, straight or flexuous, cylindrical, thick-walled, smooth, septate, brown at the base, colorless towards the apex. Conidiogenous cells monoblastic, integrated, terminal, cylindrical, brown, pale brown or subhyaline, smooth or verrucose; collarette narrow or flaring. Conidia solitary, dry, apical, simple, smooth, typically oval, ellipsoidal to spherical, brown to dark brown. Conidial secession schizolytic or rhexolytic.

Notes — Baker *et al*. established *Junewangia* with *J. sphaerospora* as the type species^3^. Four *Acrodictys* sensu lato species have been placed in *Junewangia* according to morphology^3^. The holotype was deposited in Herb. AUA. No live culture of the holotype is known to exist.

**Figure 10:**
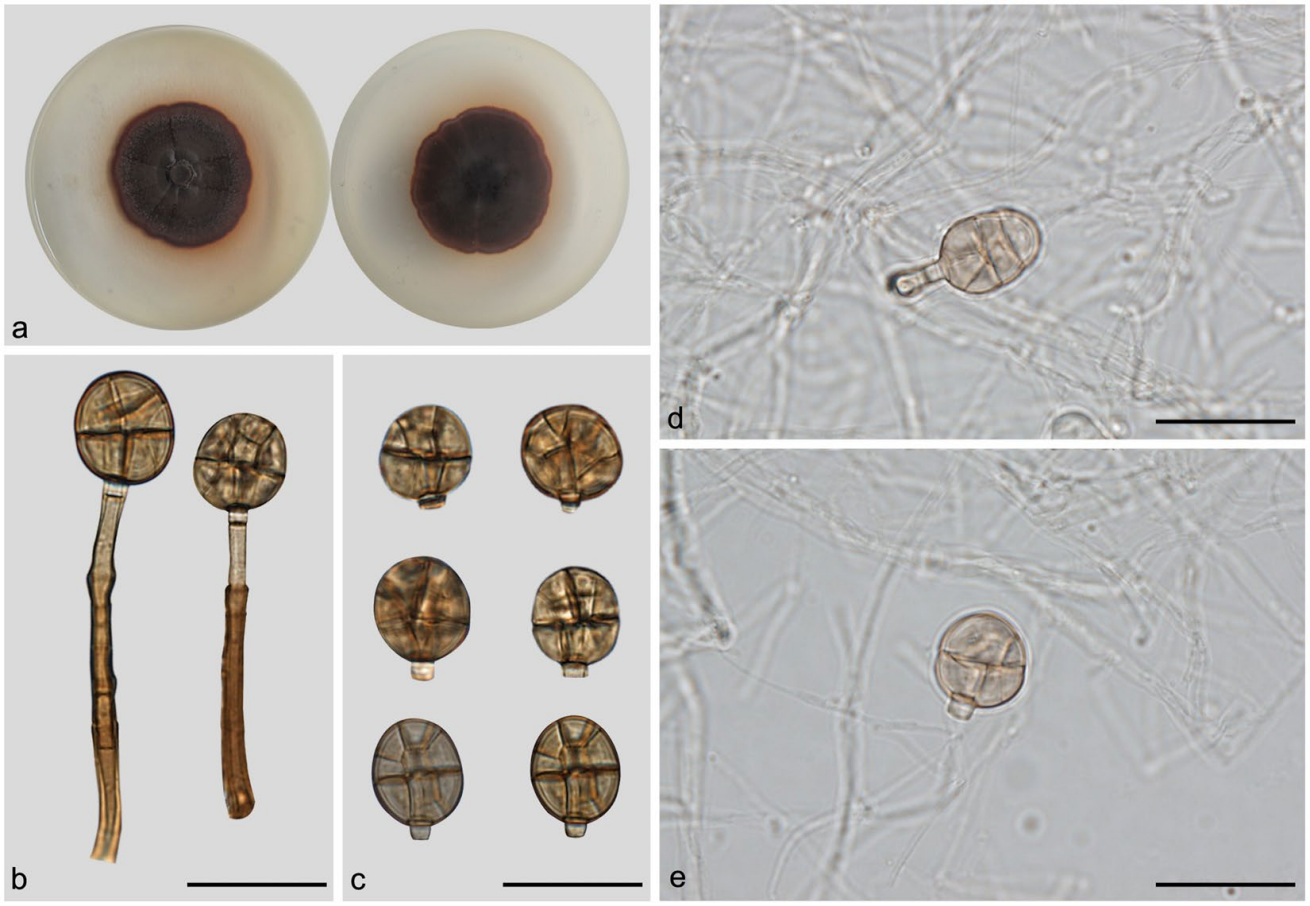
Junewangia lamma. (**a**) Colony on PDA (surface and reverse). (**b**,**c**) From natural substrate (HSAUP H4695): (**b**) Conidiophores with conidia, (**c**) Conidia. (**d**,**e**) From PDA (CGMCC 3.18652): Conidiophores and conidia. — Scale bars = 20 μm.

***Junewangia lamma*** (Whitton, McKenzie & K.D. Hyde) W.A. Baker & Morgan-Jones, Mycotaxon 81: 310. 2002. — Fig. 10.

Conidiophores scattered singly or in small groups over the substrate, erect, straight or flexuous, unbranched, cylindrical, thick-walled, smooth, brown, 1–5-septate, 8–55 μm long, 2.5–3.5 μm wide. Conidiogenous cells monoblastic, integrated, terminal, cylindrical, with 1–3 successive percurrent extensions, pale brown, smooth, truncate at the apex following conidial secession. Conidia solitary, dry, acrogenous, broadly elliptical to globose, with 1–2 transverse septa and some longitudinal or oblique septa, brown to dark brown, septa sometimes dividing the conidia cruciately, 14–18 × 12–14.5 μm, with a distinct, protruding basal cell. Conidial secession schizolytic.

Culture characteristics – Colonies on PDA, 35–40 mm diam after 14 d at 25 °C, brown near the center, reddish at the margin; reverse concolorous. No growth on MEA.

*Materials examined*. China, Yunnan Province, Xishuangbanna, Menglun Natural Reserve, 21°27′N, 100°25′E, 570 m elevation, on dead branches of an unidentified broadleaf tree, 20 Nov. 2014, Yingrui Ma, reference specimen designated here HSAUP H4695 (=HMAS 44438), living culture CGMCC 3.18652; China, Guizhou Province, Libo, Maolan National Forest Park, 25°09′N, 107°52′E, 629 m elevation, on dead branches of an unidentified broadleaf tree, 6 Nov. 2013, Yingrui Ma, reference specimen designated here HSAUP H6776 (=HMAS 245622), living culture CGMCC 3.18653.

Notes — The conidiophores and conidia of our collection are morphologically very similar to that of the type specimen^3^. *Junewangia sphaerospora* nested between two *J. lamma* (CGMCC 3.18652 and CGMCC 3.18653) in Fig. 1, but two *J. lamma* cann’t be differentiated by morphology, we think they are the same species.

**Figure 11:**
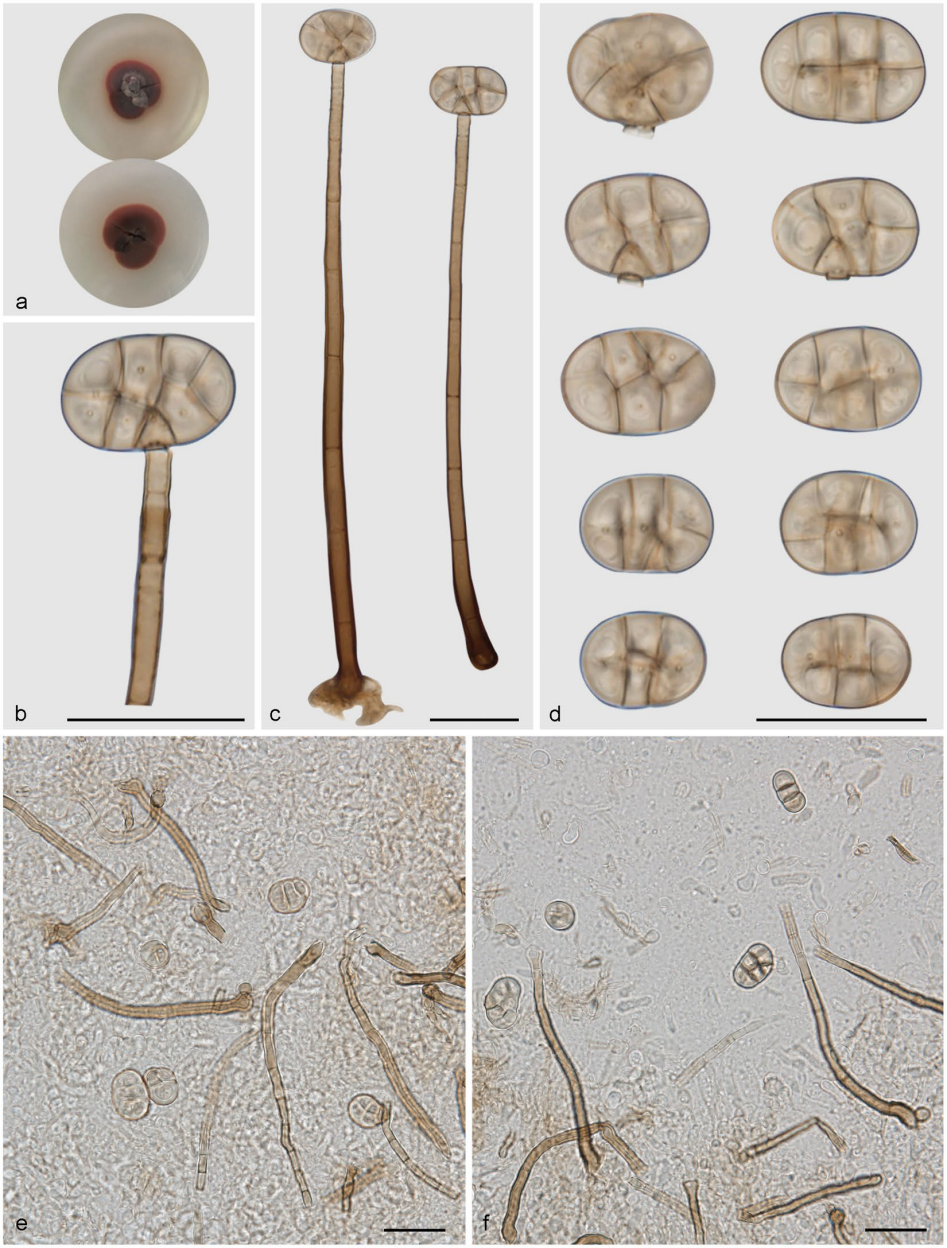
Junewangia queenslandica. (**a**) Colony on PDA (surface and reverse). (**b**–**d**) From natural substrate (HSAUP H7722): (**b**) Conidiogenous cells with conidia, (**c**) Conidiophores with conidia, (**d**) Conidia. (**e**,**f**) From PDA (CGMCC 3.18654): Conidiophores and conidia. — Scale bars = 20 μm.

***Junewangia queenslandica*** (Matsush.) J.W. Xia & X.G. Zhang, *com. nov*. – MycoBank MB 818901; — Fig. 11.

Basionym: *Acrodictys queenslandica* Matsush., Matsush. Mycol. Mem. 6: 4. 1989.

≡ *Rhexoacrodictys queenslandica* (Matsush.) W.A. Baker & Morgan-Jones, Mycotaxon 82: 110. 2002.

Conidiophores scattered singly or in groups of 2 or 3, erect, straight or flexuous, unbranched, cylindrical, thick-walled, smooth, pale brown, 5–8-septate, 103–142 × 3–4.5 μm. Conidiogenous cells monoblastic, integrated, terminal, cylindrical, with 0–2 percurrent extensions, sub-hyaline to hyaline, smooth. Conidia solitary, dry, acrogenous, elliptical or occasionally subglobose, 13–23 × 10–14.5 μm, often transverse on the conidiogenous cell, muriform, often with 1 median, transverse septum and 2–5 longitudinal or oblique septa, subhyaline to pale brown, septa sometimes dividing the conidia cruciately. Conidial secession rhexolytic.

Culture characteristics – Colonies on PDA, 30–35 mm diam after 14 d at 25 °C, pale brown near the center, black between center and the margin, reddish near the colony margin; reverse concolourous. Not growing on MEA.

*Materials examined*. China, Yunnan Province, Xishuangbanna, Menglun Natural Reserve, 21°27′N, 100°25′E, 570 m elevation, on dead branches of an unidentified broadleaf tree, 20 Nov. 2014, Xiangyu Li, reference specimen designated here HSAUP H7722 (=HMAS 245634), living culture CGMCC 3.18654.

Notes — We compared HMAS 245634 with the original description of *Acrodictys queenslandica* by Matsushima, the specimen HMAS 245634 fits well with the original description^16^. What’s more, the specimens of Matsushima and us are come from dead branches of broadleaf tree^16^. Therefore, we justify that our specimen HMAS 245634 could represent the species *Acrodictys queenslandica*. Baker *et al*. transferred this species to *Rhexoacrodictys* based on the percurrently extending conidiophores and rhexolytically seceding conidia^4^. However, CGMCC 3.18654 clustered in the *Junewangia* clade in Fig. 1. We treat this taxon as a new combination in the genus *Junewangia*, *J. queenslandica*.

**Figure 12:**
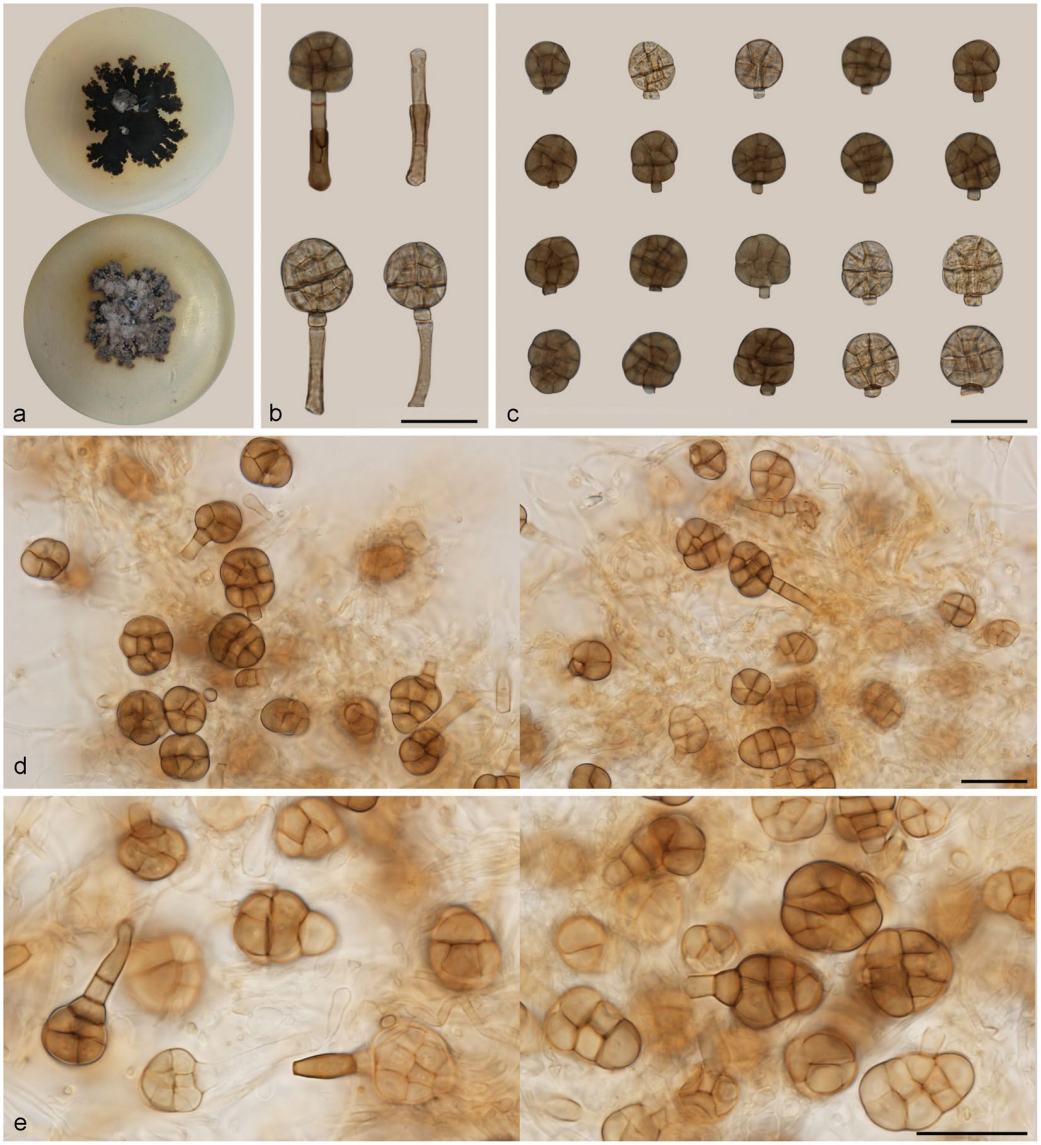
Junewangia sphaerospora. (**a**) Colony on PDA (surface and reverse). (**b**,**c**) From natural substrate (HSAUP H4733): (**b**) Conidiophores, conidiogenous cells and conidia, (**c**) Conidia. (**d**,**e**) From PDA (CGMCC 3.18655): Conidiophores and conidia. — Scale bars = 20 μm.

***Junewangia sphaerospora*** W.A. Baker & Morgan-Jones, Mycotaxon 81: 312. 2002. — Fig. 12.

Conidiophores macronematous, mononematous, single or in small groups, erect, unbranched, straight or flexuous, cylindrical, thick-walled, smooth, swollen at the base, aseptate or sometimes with 1–4 septa where regenerative growth occurs, up to 35 μm long, 2.5–4 μm wide at the broadest part, olivaceous brown to pale brown. Conidiogenous cells monoblastic, integrated, terminal, cylindrical, with 1–5 percurrent extensions, pale olivaceous brown, smooth, truncate at the apex following conidial secession. Conidia solitary, dry, acrogenous, spherical to subspherical, with 1–2 inconspicuous transverse septa and numerous oblique septa, slightly constricted at the septa, olivaceous brown to dark brown, 12–22 × 12–18 μm, often slightly wider than tall; base distinctly protuberant, truncate. Conidial secession schizolytic.

Culture characteristics – Colonies on PDA, 25–30 mm diam after 14 d at 25 °C, pale brown to reddish brown, mycelium sparse; reverse blackish. No growth on MEA.

*Materials examined*. China, Hainan Province, Qiongzhong, Limushan Natural Reserve, 19°16′N, 109°38′E, 959 m elevation, on dead branches of an unidentified broadleaf tree, 26 Apr. 2014, Yingrui Ma, reference specimen designated here HSAUP H4733 (=HMAS 245631), living culture CGMCC 3.18655.

Notes — *Junewangia sphaerospora* is distinct in this genus by its olivaceous coloration and the lack of conidiophore septation^3^. Compared with the original description, our collection has shorter conidiophores (35 μm vs 65 μm), but they are similar in conidial shape and pigmentation. This is the first record of this species from China.

***Distoseptisporaceae*** K.D. Hyde & McKenzie, Fungal Diversity 80: 402. 2016.

*Type genus*. *Distoseptispora* K.D. Hyde, McKenzie & Maharachch., Fungal Diversity 80: 402. 2016.

Description^29^.

Notes — The family *Distoseptisporaceae*, established with strong molecular support, was monotypic for *Distoseptispora* ^29^.

***Distoseptispora*** K.D. Hyde, McKenzie & Maharachch., Fungal Diversity 80: 402. 2016.

*Type species*. *Distoseptispora fluminicola* McKenzie, H.Y. Su, Z.L. Luo & K.D. Hyde, Fungal Diversity 80: 402. 2016.

Description^29^.

Notes — The genus *Distoseptispora* was erected by *D. fluminicola* based on both morphology and molecular data^29^.

**Figure 13:**
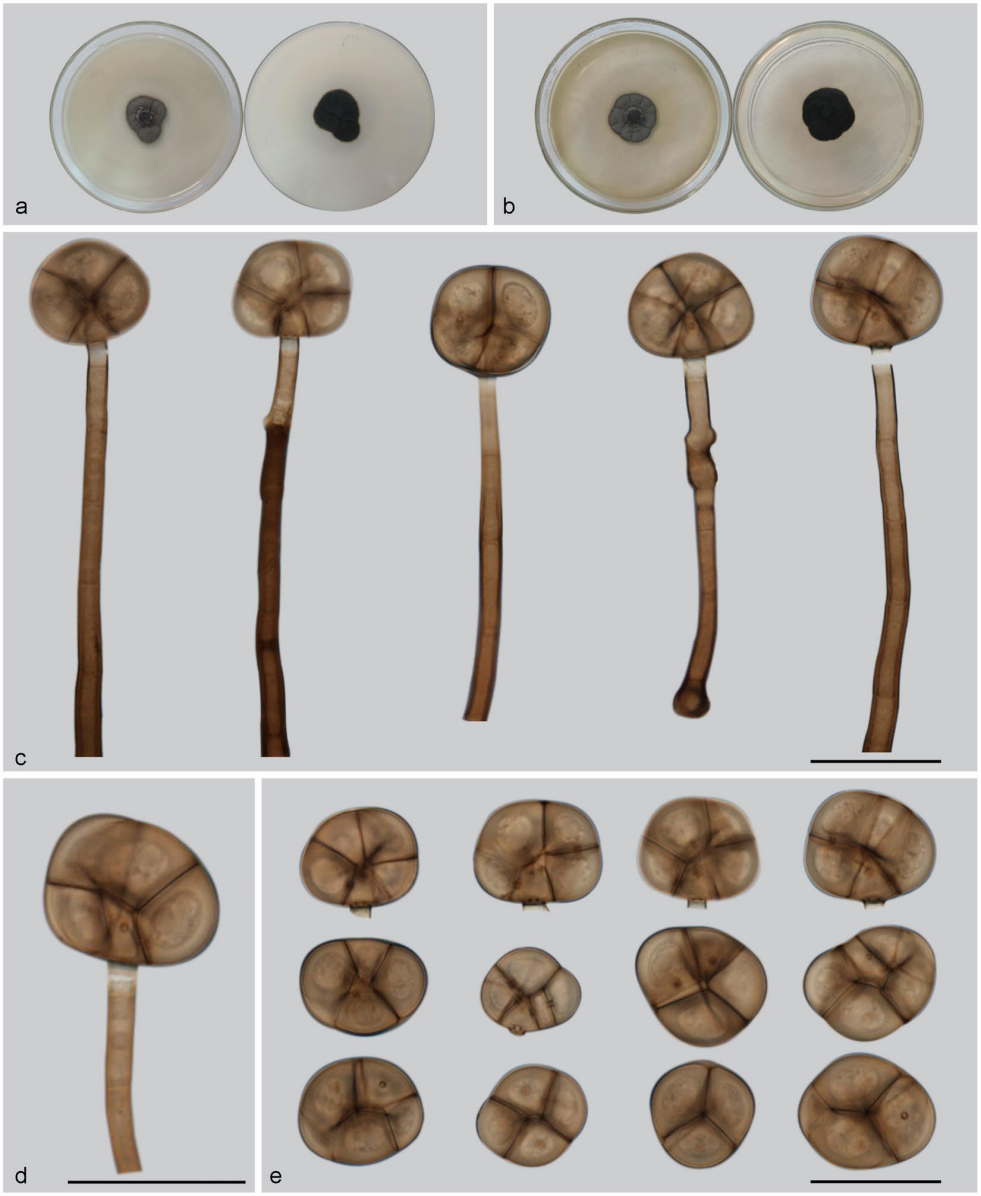
Distoseptispora martini. (**a**) Colony on PDA (surface and reverse). (**b**) Colony on MEA (surface and reverse). (**c**–**e**) From natural substrate (HSAUP H4280): (**c**) Conidiophores with conidia, (**d**) Conidiogenous cells with conidia. (**e**) Conidia. — Scale bars = 20 μm.

***Distoseptispora martinii*** (J.L. Crane & Dumont) J.W. Xia & X.G. Zhang, *comb. nov*. – MycoBank MB 821621; — Fig. 13.

Basionym: *Acrodictys martinii* J.L. Crane & Dumont, Can. J. Bot. 53: 846. 1975.

≡ *Junewangia martinii* (J.L. Crane & Dumont) W.A. Baker & Morgan-Jones, Mycotaxon 81: 310. 2002.

≡ *Rhexoacrodictys martinii* (J.L. Crane & Dumont) G. Delgado, Mycotaxon 107: 369. 2009.

Conidiophores macronematous, mononematous, solitary or in groups of a few, erect, unbranched, straight or flexuous, cylindrical, thick-walled, smooth, 4–9-septate, 50–110 μm long, 3.5–4.5 μm wide, dark brown for the most part, paler towards the apex. Conidiogenous cells monoblastic, integrated, terminal, cylindrical, with 0–2 percurrent extensions, subhyaline to pale brown, smooth. Conidia solitary, dry, transversal ellipsoid, oblate or subglobose, 15–20 × 11–16 μm, muriform, appearing divided cruciately by septa at right angles to one another, sometimes with pores in the septa, pale brown to brown. Conidial secession rhexolytic.

Culture characteristics – Colonies on PDA, 20–25 mm diam after 14 d at 25 °C, dark brown above, mycelium sparse; reverse blackish. Colonies on MEA, 20–25 mm diam after 14 d at 25 °C, margin regular, dark brown above; reverse blackish.

*Materials examined*. China, Guizhou Province, Libo, Maolan National Forest Park, 25°09′N, 107°52′E, 629 m elevation, on dead branches of an unidentified broadleaf tree, 6 Nov. 2013, Yingrui Ma, reference specimen designated here HSAUP H4280 (=HMAS 245625), living culture CGMCC3.18651.

Notes — This fungus was first described as *Acrodictys martinii* by Crane & Dumont, based on a Puerto Rican collection^9^. Baker *et al*. transferred the species to *Junewangia* W.A. Baker & Morgan-Jones because of its percurrent extending conidiogenous cells and subspherical to almost spherical conidia^3^. Delgado’s study of type and new collections from Florida revealed rhexolytic secession, a defining character of *Rhexoacrodictys* W.A. Baker & Morgan-Jones^32^. Accordingly, Delgado transferred the species to *Rhexoacrodictys*. According to morphology and phylogenetic analysis (Fig. 1), we treat this species as a new combination as *Distoseptispora martinii*.

***Savoryellaceae*** Jaklitsch & Réblová, Index Fungorum 2015: 209. 2015.

*Type genus*. *Savoryella* E.B.G. Jones & R.A. Eaton, Trans. Br. mycol. Soc. 52: 161. 1969.

Description^33^.

Notes — The family *Savoryellaceae* was introduced by Jaklitsch and Réblová^33^. Asexual morphs are dematiaceous hyphomycetes, e.g., *Canalisporium* linked to *Ascothailandia* and *Monotosporella*, and *Helicoon* linked to *Ascotaiwania*. Species of *Savoryellaceae* are predominantly found in aquatic habitats such as freshwater, marine and brackish environments, particularly on submerged wood^33^.

***Rhexoacrodictys*** W.A. Baker & Morgan-Jones, Mycotaxon 82: 98. 2002.

*Type species*. *Rhexoacrodictys erecta* (Ellis & Everh.) W.A. Baker & Morgan-Jones, Mycotaxon 82: 99. 2002.

Colonies effuse, brown, hairy. Mycelia partly superficial, partly immersed in the substrate. Sexual morph: Undetermined. Asexual morph: Hyphomycetous. Conidiophores macronematous, mononematous, erect, unbranched, straight or flexuous, thick-walled, smooth, dark brown at the base, paler and narrower towards the apex, septate. Conidiogenous cells monoblastic, integrated, terminal, pale brown, smooth, cylindrical, with percurrent extensions. Conidia solitary, dry, acrogenous, broad ellipsoidal, obpyriform or obovoid, muriform, often with multiple transverse and longitudinal or oblique septa, slightly constricted at septa, basal cell protruding, cylindrical, often with a marginal frill following secession. Conidial secession rhexolytic.

Notes — Baker *et al*. established *Rhexoacrodictys* with *R. erecta* as the type species^4^. And four *Acrodictys* sensu lato species were placed in *Rhexoacrodictys* according to morphology^4^. *Rhexoacrodictys* clustered in the *Savoryellaceae* clade in Fig. 1.

**Figure 14:**
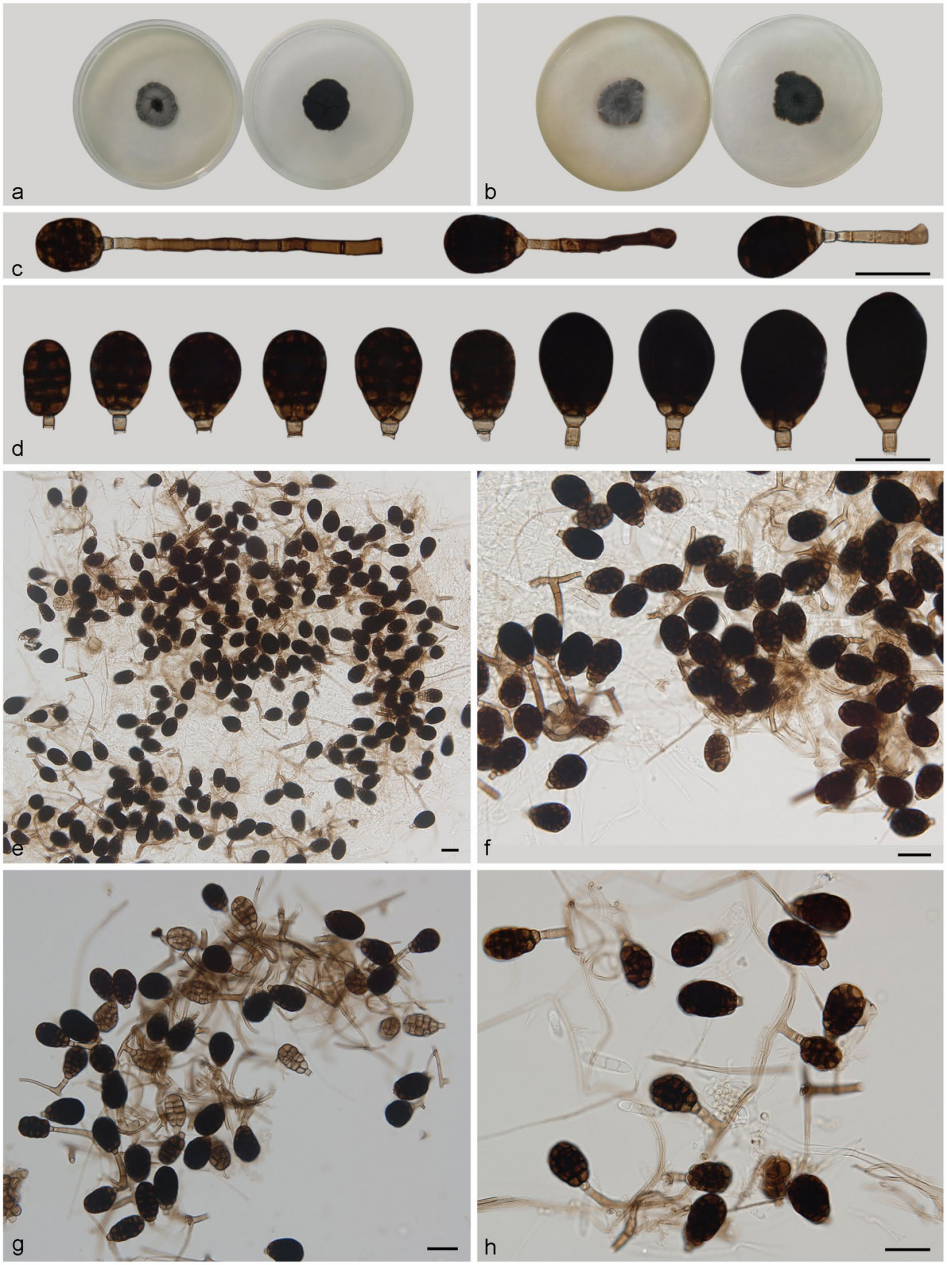
Rhexoacrodictys erecta. (**a**) Colony on PDA (surface and reverse). (**b**) Colony on MEA (surface and reverse). (**c**,**d**) From natural substrate (HSAUP H4622): (**c**) Conidiophores, Conidiogenous cells and conidia, (**d**) Conidia. (**e**–**h**) From PDA (CGMCC 3.18656): Conidiophores and conidia. — Scale bars = 20 μm.

***Rhexoacrodictys erecta*** (Ellis & Everh.) W.A. Baker & Morgan-Jones, Mycotaxon 82: 99. 2002. — Fig. 14.

Basionym: *Mystrosporium erectum* Ellis & Everh., J. Mycol. 4: 53. 1888.

≡ *Macrosporium erectum* (Ellis & Everh.) Pound & Clem., Minn. Bot. Stud. 9: 657. 1896.

≡ *Acrodictys erecta* (Ellis & Everh.) M.B. Ellis, Mycol. Pap. 79: 12. 1961.

=*Piricauda serendipita* R.T. Moore, Rhodora 61: 104. 1959.

=*Acrodictys satwalekeri* D. Rao, Curr. Sci. 5: 117. 1970.

Conidiophores 40–75 μm long, macronematous, mononematous, erect, unbranched, straight or flexuous, thick-walled, smooth, dark brown at the base, 3.5–5 μm wide, paler and narrower towards the apex, septate. Conidiogenous cells monoblastic, integrated, terminal, pale brown, smooth, cylindrical, with percurrent extensions. Conidia solitary, dry, acrogenous, turbinate, broad ellipsoidal, obpyriform or obovoid, 20–40 × 12.5–22 μm, muriform, often with multiple transverse and longitudinal or oblique septa, slightly constricted at septa, mature conidia dark blackish in the upper 3/4, paler for the lower 1/4; basal cell protruding, cylindrical, often with a marginal frill following secession. Conidial secession rhexolytic.

Culture characteristics – Colonies on PDA, 40–45 mm diam after 14 d at 25 °C, brown in the front side, blackish at the reverse side, mycelium sparse; reverse blackish. Colonies on MEA, 40–45 mm diam after 14 d at 25 °C, margin regular, brown in the front side, blackish at the reverse side; reverse blackish.

*Materials examined*. China, Hainan Province, Lingshui, Diaoluoshan National Forest Park, 18°42′N, 108°52′E, 1499 m elevation, on dead branches of an unidentified broadleaf tree, 23 Apr. 2014, Yingrui Ma, Jiwen Xia, reference specimen designated here HSAUP H4622 (=HMAS 245615), HSAUP H6489 (=HMAS 245616), living culture CGMCC 3.18656, CGMCC 3.18657.

Notes — Ellis reported this species on *Arundo donax* in Venezuela and on *Zea mays* in USA^1^. Baker *et al*. examined several type specimens of synonyms of this species and erected *Rhexoacrodictys*, with *R. erecta* as the type species, on the basis of conidial morphology and detachment process^4^. The characters of our specimens fit well with the concept of the genus and morphologies of *Rhexoacrodictys erecta*.

**Figure 15:**
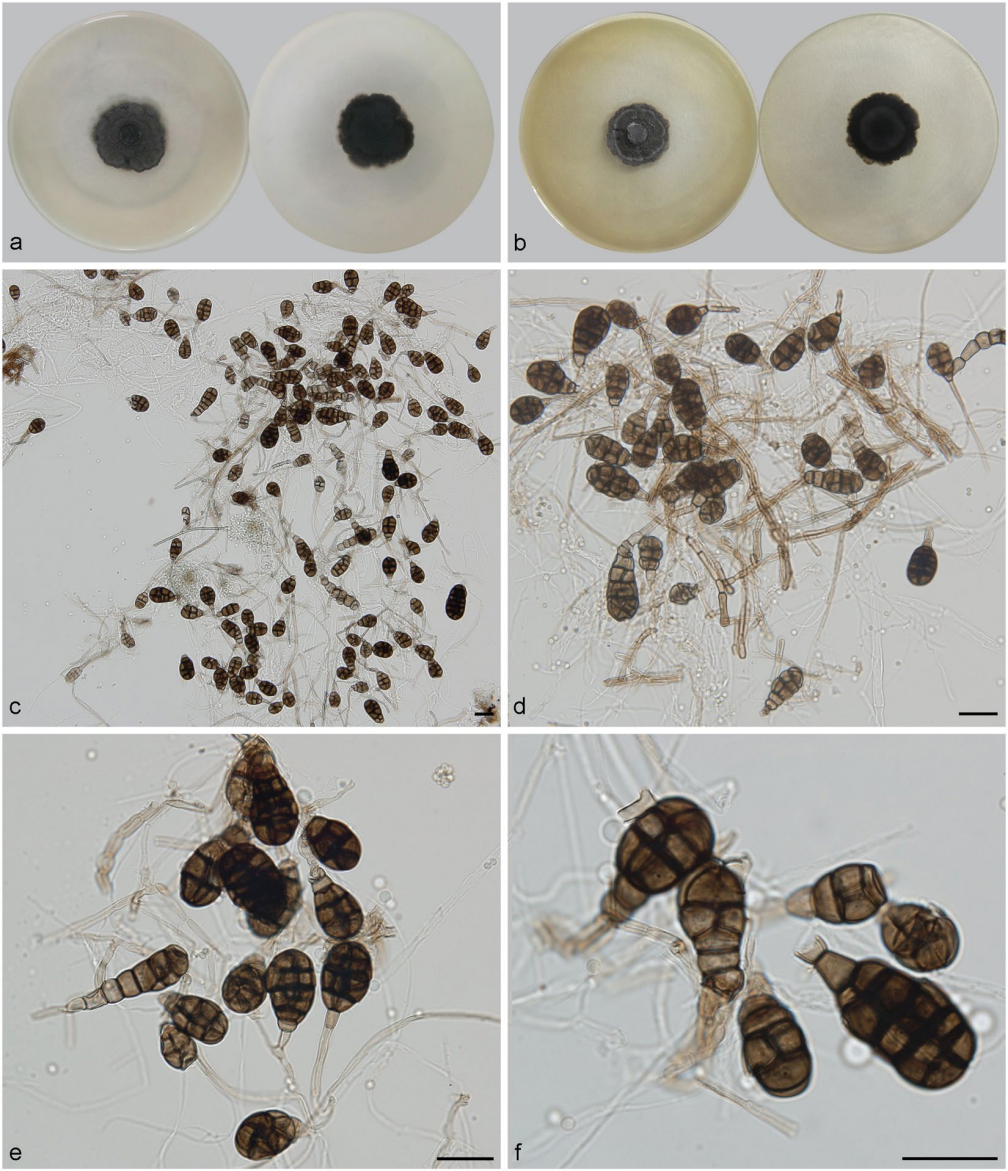
Rhexoacrodictys fimicola. (**a**) Colony on PDA (surface and reverse). (**b**) Colony on MEA (surface and reverse). (**c**–**f**) From PDA (CGMCC 3.18658): Conidiophores and conidia. — Scale bars = 20 μm.

***Rhexoacrodictys fimicola*** (M.B. Ellis & Gunnell) W.A. Baker & Morgan-Jones, Mycotaxon 82: 103. 2002. — Fig. 15.

Basionym: *Acrodictys fimicola* M.B. Ellis & Gunnell, Mycol. Pap. 79: 10. 1961.

=*Acrodictys sacchari* M.B. Ellis, Mycol. Pap. 125: 6. 1971.

Conidiophores up to 45 μm long in culture, sometimes in the form of chains of thick-walled, brown, torulose chlamydospore-like cells, pale brown to brown. Conidiogenous cells monoblastic, integrated, terminal, percurrent extensions not observed. Conidia obovoid, 22–44 × 11–20 μm, muriform, usually with 3–5 transverse septa and several longitudinal or oblique septa, dark brown; basal cell protruding, cylindrical, subhyaline to pale brown. Conidial secession rhexolytic.

Culture characteristics – Colonies on PDA, 30–35 mm diam after 14 d at 25 °C, surface dark brown, mycelium sparse; reverse blackish. Colonies on MEA, 25–30 mm diam after 14 d at 25 °C, margin regular, surface dark brown, reverse blackish.

*Materials examined*. China, Hainan Province, Lingshui, Diaoluoshan National Forest Park, 18°42′N, 108°52′E, 1499 m elevation, on dead branches of an unidentified broadleaf tree, 23 Apr. 2012, Wenping Wu, reference specimen designated here HMAS 47737, HMAS 43690, living culture CGMCC 3.18658, CGMCC 3.18659; China, Yunnan Province, Xishuangbanna, Menglun Nature Reverse, 21°27′N, 100°25′E, 552 m elevation, on dead branches of an unidentified bamboo, 22 Nov. 2015, Jiwen Xia, reference specimen designated here HMAS 42882, living culture CGMCC 3.18660.

Notes — Conidiophores described in the protologue are longer (up to 80 μm) than were observed in our culture (up to 45 μm) and the conidia and conidiophores produced in culture are lighter than that originally described and illustrated.

## Discussion

Baker *et al*. and Baker & Morgan-Jones divided the genus *Acrodictys* into four genera, including *Acrodictys* sensu stricto, *Junewangia*, *Pseudoacrodictys* and *Rhexoacrodictys* ^2–4^. Seifert et al. provided a key to morphologically defined genera that are characterized by the formation of conidiomata and lobbed conidia^7^. In the present work we focus on genera that do not have conidiomata or lobbed conidia. These genera share the basic morphology of conidiophores that are macronematous, mononematous, cylindrical and unbranched or infrequently branched. There is only one conidiogenous cell and it is integrated into the tip of the conidiophore. The conidiogeous cell produces a single blastic conidium, after which it extends percurrently one or more times, producing a single conidium with each extension. Conidia are essentially bicellular but the apical cell, which is larger and typically subglobose, becomes darkly pigmented and muriform, with various numbers of transverse and longitudinal septa. The basal cell is typically only lightly pigmented and much smaller than the upper cell; it is conspicuous or not, and flat at the point of secession from the conidiogenous cell. Conidia of *Ityorhoptrum* remain bicellular. The basal cells in *Acrodictys* and *Rhexoacrodictys* are conspicuous, cuneiform or funnel shaped, while the basal cells in *Junewangia* is inconspicuous, reduced to a short, cylindrical protrusion from the apical cell.

These fungi are easily recognized as a morphological group but when compared side-by-side one could question whether the described characters indicate phylogenetically meaningful genera. To test this hypothesis, we utilized phylogenetic analysis based on DNA sequences. From our study we cannot say that the discussed genera actually form a monophyletic group. The multi-gene analyses (Fig. 1) indeed placed the type species of *Acrodictys*, *Junewangia* and *Rhexoacrodictys* in distinct clades. The family *Acrodictyaceae* and *Junewangiaceae* were established to accommodate the genera *Acrodictys* and *Junewangia*, respectively. The genera *Distoseptispora* and *Rhexoacrodictys* belong to the family *Distoseptisporaceae* and *Savoryellaceae*, respectively. The present study provides a backbone tree for these four families.

*Junewangia queenslandica* (CGMCC 3.18654), which clustered in the *Junewangiaceae* clade (Fig. 1), previously was identified as *Rhexoacrodictys* on the basis of its morphology. *Distoseptispora martini* (CGMCC 3.18651), previously has been identified as *Rhexoacrodictys* on the basis of its morphology, but the phylogenetic analyses indicated that this species is not closely related to *Rhexoacrodictys* but clusters in the *Distoseptisporaceae* clade (Fig. 1).

Traditionally, Acrodictys-like species have been characterised and identified based on conidial schizolytic/rhexolytic secession, and morphology of conidiophores, conidiogenous cells and conidia^2–4, 7^. All species having rhexolytic conidial dehiscense have been placed in *Rhexoacrodictys* but the multi-gene phylogenetic tree shows that they are not all congeneric. *Junewangia queenslandica* (CGMCC 3.18654) and *Distoseptispora martini* (CGMCC 3.18651) previously have been identified as *Rhexoacrodictys* species because of their rhexolytic conidial dehiscense, but they clustered in the *Junewangiaceae* and *Distoseptisporaceae* respectively. Results from the present study revealed that conidial schizolytic/rhexolytic secession in Acrodictys-like species are not informative as generic characters, but appeared to be highly informative at the species level.

## Materials and Methods

### Specimen examination and isolation

The specimens were collected from five locations in three provinces in southern China (Hainan, Yunnan, Guizhou), and taken to the laboratory in plastic bags. The samples were processed and examined following the methods of Ariyawansa *et al*.^34^. Fresh and herbarium materials were examined using an Olympus SZX10 dissecting microscope to locate sporulating structures. Squash mounts of the sporulating structures were mounted in water for microscopic studies and photomicrography. The specimens were examined using an Olympus BX53 microscope and photographed with an Olympus DP73 digital camera (Olympus, Japan) fitted to the microscope. At least 50 mature conidia and 30 conidiophores were mounted in water, measured at Olympus cellSens software (Olympus, Japan) and images used for figures processed with Adobe Photoshop version 7.0 software (Adobe Systems, USA). Isolations were made from single conidia, following a modified method of Chomnunti *et al*.^35^. The specimens were deposited in the Herbarium of Department of Plant Pathology, Shandong Agricultural University, Taian, Shandong, China (HSAUP) and the Mycological Herbarium, Institute of Microbiology, Chinese Academy of Sciences, Beijing, China (HMAS). The living cultures were deposited in the China General Micriobiological Culture Collection centre (CGMCC).

### DNA isolation, amplification and sequencing

Total genomic DNA was extracted using the CTAB method^36^. For PCR reactions, the following primers were used: ITS: ITS5-ITS4^37^; ncSSU: 18 SF-18SR900 (This study, 18S-F: 5′-CTCGTAGTTGAAACTTGGGCC-3′, 18S-R: 5′-TTATCCCCAGCACGACAGAG-3′); ncLSU: 28S1–28S3 (This study, 28S1-F: 5′-AGTAACGGCGAGTGAAGCG-3′, 28S3-R: 5′-ACTCCTTGGTCCGTGTTTCA-3′); *tub*2: BetaF-BetaR (This study, Beta-F: 5′-GGTAACCAAATCGGTGCTGC-3′, Beta-R: 5′-ACCCTCGGTGTAGTGACCCTT-3′). Reaction mixtures contained 5 μL of 10 × ThermoPol reaction buffer [200 mM Tris-HCl, pH 8.3, 100 mM KCl, 100 mM (NH_4_)_2_SO_4_, 20 mM MgSO_4_ and 1% Triton X-100], 20 ng template genomic DNA, 2 pmol of each primer, 4 μL of 2.5 mM dNTPs, 0.5 U of AmpliTaq polymerase, and total volume was adjusted to 50 μL with deionized water. The PCR thermal cycle for ncSSU, ncLSU and ITS region amplification was as follows: 94 °C for 5 min, followed by 35 cycles of denaturation at 94 °C for 30 s, annealing at 57 °C for 50 s and elongation at 72 °C for 90 s, with a final extension step of 72 °C for 10 min. The PCR thermal cycle for *tub2* region amplication was as follows: 94 °C for 3 min, followed by 35 cycles of denaturation at 94 °C for 30 s, annealing at 57 °C for 30 s and elongation at 72 °C for 50 s, with a final extension step of 72 °C for 10 min. The PCR-amplified DNA fragments were fractionated in 1.0% agarose gels in 0.5 × TBE buffer, and reveled under UV illumination. The PCR products were purified using a DNA fragment Purification Recovery Kit (BioTeke, China). Sequencing of both strands of each fragment was performed with an ABI PRISM 3730 DNA autosequencer using either dRhodamine terminator or Big Dye Terminator chemistry (Applied Biosystems, Foster City, CA, USA). The DNA sequences of ncLSU, ncSSU, ITS and *tub2* genes generated in this study were submitted to GenBank (www.ncbi.nlm.nih.gov).

### Phylogenetic analyses

Sequences from each primer combination were used to obtain consensus sequences with MEGA v. 6.0^38^. Reference sequences from Maharachchikumbura *et al*. and Su *et al*. were downloaded from GenBank, and are listed in Table S1 ^28, 29^. Alignments of all consensus sequences, as well as the reference sequences were generated with MAFFT v. 7 (http://mafft.cbrc.jp/alignment/server/index.html)^39^, and were improved manually when necessary. Ambiguous regions were excluded from the analyses and gaps were treated as missing data. A 70% neighbour-joining (NJ) reciprocal bootstrap method with maximum-likelihood distance was applied to check the congruence of the individual loci in the multi-locus dataset^40^. Phylogenetic analyses of both individual and combined aligned data consisted of Bayesian and maximum-likelihood analyses.

Phylogenetic reconstruction utilized Bayesian inference (BI) running on MrBayes v.3.2.2^41^ by automatically sampling across the entire general time-reversible (GTR) substitution model space in the Bayesian MCMC analysis itself^42^. The number of generations was set at 10 million and the run was stopped automatically when the average standard deviation of split frequencies fall below 0.01. Trees were saved each 1 000 generations. Burn-in was set at 25% after which the likelihood values were stationary and the remaining trees were used to calculate posterior probabilities. Maximum-likelihood analyses including 1 000 bootstrap replicates were conducted using RAxML v. 7.2.6^43^. A general time reversible model (GTR) was applied with a gamma-distributed rate variation. The final matrices used for phylogenetic analyses in TreeBASE (www.treebase.org; accession number: 20157), and novel taxonomic descriptions and nomenclature in MycoBank (www.MycoBank.org)^44^.

## Electronic supplementary material

Supplementary information
