# Supplementary material for: Acrodictys-like wood decay fungi from southern China, with two new families Acrodictyaceae and Junewangiaceae
Source: Sci Rep. 2017 Aug 11;7:7888. doi: 10.1038/s41598-017-08318-x (PMC5554248; doi:10.1038/s41598-017-08318-x)
Supplement: Supplementary file 1 — Supplementary information [file 41598_2017_8318_MOESM1_ESM.pdf]

Acrodictys-like wood decay fungi from southern China, with two new families *Acrodictyaceae* and *Junewangiaceae*

Ji Wen Xia, Ying Rui Ma, Zhuang Li* & Xiu Guo Zhang*

* Corresponding author: E-mail sdau613@163.com; zhxg@sdau.edu.cn.

Shandong Provincial Key Laboratory for Biology of Vegetable Diseases and Insect Pests, College of Plant Protection, Shandong Agricultural University, Taian, 271018 Shandong, China

Supplementary Table S1. List of taxa used in this study and their GenBank accession numbers.

Taxa	Source*	GenBank accession numbers			
		LSU	SSU	ITS	*tub2*
Acrodictys bambusicola	CGMCC 3.18641	KX033564	KX033535	KU999973	KX036219
Acrodictys elaeidicola	CGMCC 3.18642	KX033568	KX033539	KU999977	–
	CGMCC 3.18643	KX033569	KX033540	KU999978	–
Acrodictys globulosa	CGMCC 3.18644	KX033562	KX033532	KU999970	–
Acrodictys hainanensis	CGMCC 3.18645	KX033565	KX033536	KU999974	KX036218
Acrodictys liputii	CGMCC 3.18646	KX033558	KX033528	KU999966	–
	CGMCC 3.18647	KX033570	KX033541	KU999979	–
Acrodictys malabarica	CGMCC 3.18648	KX033560	KX033530	KU999968	–
Acrodictys peruamazonensis	CGMCC 3.18649	KX033561	KX033531	KU999969	–
Acrodictys porosiseptata	CGMCC 3.18650	KX033559	KX033529	KU999967	KX036220
Annulusmagnus triseptatus	CBS 128831	GQ996540	JQ429242	–	–
Annulatascus velatisporus	HKUCC 3701	AF132320	–	–	–
Ascotaiwania lignicola	NIL 00005	HQ446364	HQ446284	HQ446341	–
Ascothailandia grenadoidia	SS 03615	GQ390267	GQ390252	GQ390282	–
Botrytinia fuckeliana	AFTOL-ID 59	AY544651	AY544695	DQ491491	–
Buergenerula spartinae	ATCC 22848	DQ341492	DQ341471	JX134666	–
Canalisporium caribense	SS 03839	GQ390268	GQ390253	GQ390283	–
Carpoligna pleurothecii	CBS 114211	JQ429235	JQ429249	JQ429156	–
Ceratocystiopsis minuta	CBS 116963	EU913655	–	EU913696	EU913735
Coniochaeta ligniaria	NRRL 30616	AY198388	–	AY198390	–
Conioscyphascus varius	CBS 113653	AY484512	–	–	–
Cordana pauciseptata	CBS 121804	HE672160	–	HE672149	–
Cryptadelphia groenendalensis	SH 12	EU528007	–	–	–
	SMH 3767	EU528001	–	–	–
Distoseptispora adscendens	HKUCC 10820	DQ408561	–	–	–
Distoseptispora leonensis	HKUCC 10822	DQ408566	–	–	–
Distoseptispora martinii	CGMCC 3.18651	KX033566	KX033537	KU999975	KX036217
Dothidea sambuci	DAOM 231303	AY544681	AY544722	AY883094	–
Fragosphaeria purpurea	CBS 133.34	AB189154	AF096176	–	–
Junewangia lamma	CGMCC 3.18652	KU751882	KX033523	KU999961	–
	CGMCC 3.18653	KU751883	KX033533	KU999971	–
Junewangia queenslandica	CGMCC 3.18654	KX033575	KX033546	KU999984	KX036212

Taxa	Source*	GenBank accession numbers			
		LSU	SSU	ITS	*tub2*
Junewangia sphaerospora	CGMCC 3.18655	KX033572	KX033543	KU999981	KX036215
Lecythophora luteoviridis	CBS 206.38	FR691987	–	HE610333	–
Magnaporthe salvinii	ATCC 44754	JF414887	JF414862	JF414838	–
Ophiostoma piliferum	AFTOL-ID 910	DQ470955	DQ471003	–	–
Papulosa amerospora	AFTOL-ID 748	DQ470950	DQ470998	–	–
Pleurothecium semifecundum	CBS 131271	JQ429240	JQ429254	JQ429159	–
Raffaelea canadensis	CBS 168.66	EU177458	EU170270	–	–
Rhexoacrodictys erecta	CGMCC 3.18656	KX033555	KX033525	KU999963	KX036223
	CGMCC 3.18657	KX033556	KX033526	KU999964	KX036222
Rhexoacrodictys fimicola	CGMCC 3.18658	KX033553	KX033522	KU999960	KX036225
	CGMCC 3.18659	KX033550	KX033519	KU999957	KX036228
	CGMCC 3.18660	KX033554	KX033524	KU999962	KX036224
Savoryella lignicola	NF 00204	HQ446378	HQ446300	–	–
Sporidesmium aquaticum	MFLUCC 15-0420	KU376273	–	–	–
Sporidesmium bambusicola	HKUCC 3578	DQ408562	–	–	–
Sporidesmium fluminicola	MFLUCC 15-0346	KU376271	–	–	–
Sporidesmium minigelatinosa	NN 47497	DQ408567	–	–	–
Sporidesmium parvum	HKUCC 10836	DQ408558	–	–	–
Sporidesmium sp.	HKUCC10558	DQ408565	–	–	–
Sporidesmium submersum	MFLUCC 15-0421	KU376272	–	–	–
Thyridium vestitum	AFTOL-ID 172	AY544671	AY544715	–	–

*AFTOL: Assembling the Fungal Tree of Life, Minnesota, USA; ATCC: American Type Culture Collection, Virginia, USA; CBS: Centraalbureau voor Schimmelcultures, Utrecht, The Netherlands; CGMCC: China General Micriobiological Culture Collection centre, Beijing, China; DAOM: Canadian Collection of Fungal Cultures, Ottawa, Canada; HKUCC: The University of Hong Kong Culture Collection, Hong Kong, China; MFLUCC: Mae Fah Luang University Culture Collection, Thailand; NN, Novozymes China Culture Collection, Beijing, China; NRRL: American Research Service (ARS) culture collection, Beltsville, MD, USA; SH, SMH: S.M. Huhndorf; Isolates with the prefix SS, NF and NIL are from the BIOTEC Culture Collection (BCC).
